# Subcellular Fractionation Enables Assessment of Nucleotide Sugar Donors Inside the Golgi Apparatus as a Prerequisite for Unraveling Culture Impacts on Glycoforms of Antibodies

**DOI:** 10.1002/biot.202400678

**Published:** 2025-03-24

**Authors:** Niklas Regett, Marcel Dieterle, Fleur Peters, Max Deuring, Kaja Stegmaier, Attila Teleki, Ralf Takors

**Affiliations:** ^1^ Institute of Biochemical Engineering University of Stuttgart Stuttgart Baden‐Württemberg Germany

**Keywords:** antibody glycosylation, Golgi apparatus, metabolomics, nucleotide sugar donors, subcellular fractionation

## Abstract

Glycosylation is a critical quality attribute in biopharmaceuticals that influences crucial properties, such as biological activity and blood clearance. Current methods for modeling glycosylation typically rely on imprecise or limited data on nucleotide sugar donor (NSD) dynamics. These methods use in vitro transporter kinetics or flux balance analysis, which overlook the key aspects of metabolic regulation. We devised an integrative workflow for absolute subcellular NSD quantification in both cytoplasm and secretory organelles. Using subcellular fractionation, exhaustive sample extraction, and liquid chromatography triple‐quadrupole tandem mass spectrometry, we accurately measured NSD concentrations ranging from 1.6 amol/cell to 3 fmol/cell.

As expected, NSD concentration profiles aligned closely with the glycan distributions on antibodies, particularly after nutrient pulsing to stimulate NSD production, showcasing method validity. This method enables empirical observation of compartment‐specific NSD dynamics. Thus, this study provides novel insights indicating that N‐glycosylation, which governs NSD supply, is primarily regulated within the Golgi apparatus (GA). This method offers a novel tool to obtain sophisticated data for a more efficient optimization of glycosylation processes in production cell lines.

## Graphical Abstract and Lay Summary

A novelty analytical workflow for quantitative investigation of transport mechanisms governing the nucleotide sugar supply from the cytoplasm to the Golgi apparatus for N‐glycosylation of antibodies in Chinese hamster ovary cells was developed. The workflow enabled compartment‐specific quantification of nucleotide sugar concentrations within the cytoplasm and the secretory pathways which aligned with nutrition‐induced changes in glycosylation patterns on produced antibodies, demonstrating the efficacy of the method. With this method, we were able to illuminate the transport mechanisms of NSDs within the cell, indicating that the supply of nucleotide sugars into the Golgi apparatus is to a large extent regulated by metabolite concentrations within the Golgi.

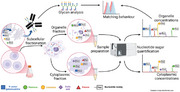

## 1 Introduction

Antibody research plays an important role in the advancement of current medicine. It offers revolutionary approaches for the treatment of a broad spectrum of diseases, including cancer and neurodegenerative and autoimmune disorders [1, 2, 3]. Antibodies are a major asset to modern medicine owing to their versatility and efficacy. Presently, antibodies are the fastest‐growing product class in the biopharmaceutical market [4]. This market is expected to experience substantial growth, with projections of up to USD 300 billion by 2025 [5]. This trajectory is driven by the critical role of antibodies in manipulating immune system activities. Their high therapeutic precision allows for the target‐specific elicitation of desired physiological effects [6].

As the demand for antibody‐based therapies increases, the biopharmaceutical industry faces both opportunities and risks. This tremendous growth is rooted in the rising prevalence of chronic diseases, such as cancer [7]. Concurrently, the industry faces increasing competitive pressure in the form of both the need for continuous approval of therapeutic agents and the impending expiration of patents for existing products [8]. Collectively, these factors underscore the need for the rapid development of new biopharmaceuticals.

Antibody glycosylation is a post‐translational modification involving the stepwise sequential attachment of complex sugar chains [9, 10, 11, 12, 13]. Gylcosylation significantly affects key features such as binding affinity, effector functionality, and pharmacokinetics [14, 15]. These factors are crucial for the therapeutic efficacy, quality, and safety. Glycosylation is an intricate mechanism that occurs predominantly in the GA. This reaction consumes nucleotide sugar donors (NSDs) derived from the cytoplasm (CP) [16, 17, 18]. Because of its reliance on monosaccharide supply, glycosylation depends on process conditions such as nutrient availability, pH, osmolarity, and temperature [19, 20, 21]. Glycosylation undergoes strict regulatory assessments during the approval process and production of biopharmaceuticals to ensure safety, efficacy, and “Quality by Design” [22].

However, current approaches do not allow quantitative and empirical access to the underlying compartment‐specific NSD transport processes [23]. The implications of this reach beyond shortcomings in targeted optimization of glycosylation precursor supply. As a result, commonly used computer modeling approaches are likely to be prone to inadequacies. These methods rely on data derived by combining whole‐cell metabolomics data with in vitro transporter kinetics data. Such approaches overlook the regulatory effects of eukaryotic cell compartmentalization because the entire NSD supply system is unknown [24]. Furthermore, inaccuracies rooted in in vitro determination of enzyme kinetic parameters can occur, as exemplified by yeast central carbon metabolism previously [25, 26].

The supply of NSDs can be experimentally measured with appropriate analytical protocols and data used to fuel comprehensive cellular models. This may reveal a sensitive correlation between the cultivation conditions, cellular activities, and antibody glycoforms. Similarly, these approaches lack access to the representative subcellular NSD data. The current state‐of‐the‐art empirical investigation of subcellular glycosylation metabolomics has significant limitations. These methods, including differential or density gradient centrifugation [27, 28, 29] and Golgi immunoprecipitation (Golgi‐IP) [30], are time‐consuming. Their dependence on temperature renders alternative methodologies more prone to metabolite degradation. Alternatively, they require genetic modifications that can potentially influence the outcomes of the research.

This study aims to refine and develop novel practices for the fractionation and quantification of metabolites in their respective organelles. This has been previously demonstrated in other compartments using subcellular metabolomics [31]. We sought to quantify NSDs in the lumens of the secretory pathway as the place of consumption and CP as the place of origin for NSDs. To obtain a more detailed temporal image, we wish to obtain direct and empirical access to the glycosylation‐governing NSD transport phenomena, overcoming the limitations of current techniques. This endeavor included novel approaches that are compatible with conventional metabolic sampling and extraction methods. It is meant to create an experimental foundation to improve glycosylation in the future and beyond, hopefully enabling the improvement of mechanistic models of antibody glycosylation along the way. Through these efforts, we aim to improve the efficiency of biopharmaceutical design and manufacturing by providing a method to support modern control strategies of glycan patterns of biopharmaceuticals, thereby facilitating compliance with regulatory assessments.

## 2 Material and Methods

### 2.1 Cultivation

Chinese hamster ovary (CHO) DP‐12 cells, of the clone #1934 (ATCC), were cultivated in shaking flasks (125/250 mL) at 37°C with 5% CO_2_ at 150 rpm agitation and saturated humidity in a shaking incubator. Viable cell density, viability, and diameter of the cells were measured daily using a fluidlab R‐300 device (anvajo GmbH). During the pulsing experiments, glucose (Glc) and lactate (Lac) levels were measured using a LaboTrace automatic analyzer (Trace Analytics GmbH). Cultivated pre‐cultures were prepared from stocks containing medium with 10% dimethyl sulfoxide (DMSO) in 125 mL shaking flasks. Subsequent primary cultures were inoculated at a concentration of 0.25 × 10^5^ cells/mL from the pre‐culture. Experiments were performed in 125 mL shaking flasks containing 30 mL of TC‐32A (XELL) medium. For the pulsing experiments, 100 mL of the same medium in 250 mL shaking flasks was used. Cultivation was conducted in triplicate.

Nutrient pulses were administered after 80.5 h with varying compositions and nutrients (Table S4). All additive solutions were prepared using the medium as a solvent and were mixed before administration.

### 2.2 Product Antibody (mAB) Quantification

mAB concentrations were determined using the enzyme‐linked immunosorbent assay (ELISA), as previously described [32].

### 2.3 Protein Fractionation

#### 2.3.1 Cell Harvest for Protein Fractionation

Suspension volumes corresponding to 7.04 × 10^6^ cells per drawn sample, were transferred to 50 mL Falcons and centrifuged at 4°C and 500 × *g* for 5 min. After decanting the supernatant, the cell pellets were washed by careful resuspension with ice‐cold phosphate‐buffered saline (PBS) buffer and subsequently aliquoted into 2 mL reaction vessels with 2 mL yielding 7.04 × 10^6^ cells for each sample. The samples were centrifuged at 4°C and 500 × *g* for 3 min and the supernatant was discarded. The entire experiment was conducted on an ice bath.

#### 2.3.2 Cell Fractionation for Proteins

Protein fractionation was performed according to Holden and Horton [33]. The fractionation (digitonin) and lysis buffers (Triton X‐114) were adjusted to the osmolarity and pH of the medium to reduce unwanted stress (Tables S1–S3). Fractionation was performed by pipetting 300 µL of fractionation buffer at varying digitonin concentrations into the reaction vessels of the washed cell pellets and incubated on ice for 10 min while gently swirling. After centrifugation at 4°C and 500 × *g* for 5 min, the supernatant was transferred to another reaction tube.

The remaining pellets were rinsed with 1 mL of ice‐cold PBS buffer and subsequently centrifuged at 4°C and 500 × *g* for 2 min. The supernatants were discarded. Lysis buffer (300 µL) was added and after centrifugation at 4°C and 3000 × *g* for 5 min, the respective supernatant was transferred to a reaction tube for storage at −70°C. Some of the remaining pellets were washed again with 300 µL of ice‐cold PBS buffer, and samples were stored after transferring the centrifuged supernatant.

#### 2.3.3 Cell Lysis of Whole Cell Samples

A combination of cell disruption methods was used to obtain whole‐cell control samples from pellets stored in liquid nitrogen immediately after the first washing step. For this, control samples were treated with a protease inhibitor (ThermoFisher), followed by Dounce homogenization (12 strokes each time per sample) in combination with a freeze‐thaw cycle, consisting of dipping the samples in liquid nitrogen (N_2_^lq^) for 10 s and thawing samples in a 100°C heat block after. This process was repeated thrice. Samples were stored at −70°C for later use.

### 2.4 Protein Quantification, Pierce

Protein concentrations were determined using a Pierce BCA Assay (Thermo Fisher Scientific, A55864) with a calibration curve of 20–2000 mg/mL bovine serum albumin standard (Thermo Fisher Scientific). The assay was performed in 96‐well plates with 210 µL working solution and 15 µL sample. Incubation took place for 1 h at 30°C with subsequent cooling to 22.5 ± 2.5°C before measurement. Absorption was measured at 562 nm using a Synergy2 96‐well plate reader (BioTek).

### 2.5 Western Blotting

#### 2.5.1 Gel Electrophoresis

Sodium dodecyl‐sulfate polyacrylamide gel electrophoresis (SDS‐PAGE) according to Laemmli [34] was used for the qualitative analysis of protein fractionation and subsequent Western blotting.

#### 2.5.2 Western Blotting

Western blot analysis was conducted using SDS‐PAGE, as previously described [35] 250X Endoplasmic reticulum western blot cocktail (Thermo Fisher Scientific) and anti‐GOLGA5 antibody were used as primary antibodies. Secondary western blot cocktail (conjugated with horseradish peroxidase [HRP]) and anti‐rabbit immunoglobulin G (IgG) secondary antibody‐HRP were used as secondary antibodies. Visualization was achieved using SuperSignal West Pico PLUS chemiluminesence substrate (ThermoFisher). Photographs were taken using a Fujifilm LAS‐3000 luminescence picture analyzer.

### 2.6 Metabolite Fractionation

#### 2.6.1 Cell Harvest for Metabolites

Based on the cell fractionation of proteins described by Holden and Horton [33] an adapted version for the cell fractionation of metabolites was developed. Cell harvesting was performed using a new procedure. For *n* samples, *n* + 1 × 8 × 10^6^ cells were harvested by centrifugation in a 50 mL Falcon at 750 × *g* and 4°C for 2 min. Pellets were resuspended in *n* + 1 × 2 mL ice‐cold PBS buffer per sample and aliquoted into 2 mL in fresh 2 mL reaction tubes. Cells were then centrifuged at 750 × *g* for 2 min at 4°C and the supernatant was discarded. Cells were harvested during the exponential growth phase after reaching a cell density between 3 and 4 × 10^6^ cells/mL, with a viability of at least 88%. Fractionation was performed immediately thereafter.

#### 2.6.2 Fractionation for Metabolites

Cell fractions were prepared using different concentrations of digitonin that were diluted from the digitonin stock solution with a diluting buffer (Table S3). Osmolarity and pH for all solutions were adjusted to be as similar as possible to the cultivation medium.

The harvested cells were resuspended in 0.3 mL fractionation buffer, followed by 7 min of incubation on ice. The suspensions were then centrifuged for 2 min at 750 × *g* and 4°C. The supernatant was transferred into a new 2 mL reaction tube, which was immediately quenched in N_2_^lq^.

The remaining pellets were washed by rinsing with 1 mL of ice‐cold PBS and subsequently centrifuged at 750 × *g* and 4°C for 2 min. The supernatant was discarded, and the remaining organelle pellets were quenched with N_2_^lq^. All samples were stored at −70°C.

#### 2.6.3 Metabolite Extraction

Metabolic extraction was performed using a modified version of the procedure developed by Bligh and Dyer [36]. Because the CP fractions were in liquid form and the organelle fractions were in pellet form, the extraction was adjusted for each type to yield comparable extracts.

In a cold room (4°C), the frozen samples were put into a −70°C cold aluminum block. Nine hundred microliter of −20°C cold extraction solution consisting of methanol/chloroform [2:1, v/v] was added, and the tubes were mixed on a vortex shaking block for 5 min. As compensation for the missing liquid volume, 300 µL of liquid chromatography‐tandem mass spectrometry (LC‐MS/MS) grade water was added to the organelle fractions, then 300 µL chloroform was added to each sample, followed by another 5 min of vortexing. The whole vortex block was then transferred to a −20°C freezer and continuously operated under this condition for 45 min, followed by 15 min of mixing at 22.5 ± 2.5°C. Afterward, the samples were centrifuged at 20,000 × *g* and 4°C for 10 min. Upper methanol/water fractions (650 µL) were transferred into a fresh 2 mL reaction tube and subsequently frozen in N_2_^lq^. The samples were then stored at −70°C until concentration.

#### 2.6.4 Lyophilization

For sample concentration using an alpha LSC basic 3–4 lyophilizer (Martin Christ), extracted samples were put into a precooled −70°C cold aluminum block in a box filled with N_2_^lq^ to keep them frozen. To allow the solvent to evaporate, holes were poured into the lids of the reaction tubes. The entire block was transferred to a lyophilizer to prevent premature thawing. The lyophilization was carried out for 24 h at 0.5 mbar and 26°C until dryness. Subsequently, samples were resealed with parafilm and stored at −70°C.

Method qualification was performed by investigating the relative quantities of extracted nucleotides because their interchangeability makes them detectable, even in the case of degradation, which can be used to quantitatively assess metabolite degradation.

#### 2.6.5 Mass Spectrometry

Targeted metabolome studies of endogenous cellular extracts were performed using an Agilent 1200 HPLC system coupled with an Agilent 6410 B triple‐quadrupole mass spectrometer with an electrospray ion source. System control and MS data acquisition were performed using the commercial MassHunter B.06.00 software (Agilent Technologies). Metabolite standards (Merck) were prepared in MS‐grade water (VWR) and stored at −70°C until use. The chromatographic separation of non‐derivatized NSDs was performed using a previously developed bicratic hydrophilic interaction chromatography (HILIC) under alkaline mobile phase conditions [37]. Samples (5 µL each) were injected onto a Sequant ZIC‐pHILIC column (150 × 2.1 mm^2^, 5 µm, Merck Millipore) with guard column (20 × 2.1 mm^2^, 5 µm, Merck Millipore). Intermediates were detected in multiple reaction monitoring mode using pre‐optimized precursor‐to‐product ion transitions (0.1 u). Internal calibration for quantification was performed using standard addition.

Internal calibration for absolute quantification was performed by standard addition to a sub‐group of samples, representative for the whole sample set. Added standard amounts were determined by previous external calibration and assessment of signal linearities of the peak areas. The resulting vial concentrations were back‐calculated to extract concentrations by considering all dilutions that occurred from sample conditioning. Extracts were based on a defined total number of cells. Shown values depict the calculated amount of analyte per cell (amol/cell) without any further assumptions. The method detection limit (MDL) for the used LC‐MS/MS method was previously defined by Teleki et al. [38]. The median MDL was at 12 nM, which is less than a third of the lowest NSD level measured in our work.

### 2.7 mAB Characterization

mABs were purified from the centrifuged supernatants obtained from the fractionation samples. Purification was performed using MabCapture ProteinA beads (ThermoFisher) with subsequent desalting, using Microsep Advance with Omega 10 kD membrane filtration units (ThermoFisher). Finally, mABs were evaporated to dryness at 30°C overnight at 4 mBar in an RVC 2–33 IR (MartinChrist).

#### 2.7.1 Enzymatic Deglycosylation With PNGase F

The glycans were enzymatically released using PNGase F. Purified mABs were dissolved in deionized water at a concentration of 1 nmol per 34.5 µL. The average molecular weight of mAB was estimated to be 150.00 kDa. Subsequently, mABs were digested using PNGaseF (ThermoFisher) under constant rotation at 400 rpm for 24 h at 50°C.

Glycan cleavage was followed by purification through filtration using ROTI Spin MINI‐10, 10 kDa MWCO membrane filters (Thermo Fisher). Firstly, membranes were washed by filtering 200 µL 70% ethanol at 13,500 × *g* until all liquid had passed through. Next, the membranes were washed twice with 400 µL deionized water and subsequently centrifuged. The samples were suspended in 400 µL with deionized water filtered. The flow‐through containing the glycans was evaporated at 30°C and 4 mbar for 4 h.

#### 2.7.2 Derivatization of Glycans

Derivatization was performed by attaching 2‐aminobenzoic acid (2‐AB) to glycans through reductive amination [39]. The labeling reagent was prepared by dissolving 50 mg/mL 2‐AB (Merck) in acetic acid and DMSO (7:3) (Merck), followed by the addition of 60 mg/mL sodium cyanoborohydride in the dark. The previously evaporated glycan samples were dissolved in 10 µL deionized water, mixed with 15 µL labeling reagent, each and incubated overnight at 37°C under light exclusion.

Labeled glycans were separated from excessive 2‐AB through size exclusion chromatography using PD MidiTrap G‐10 columns (ThermoFisher) as described by Melmer et al. [40].

Samples were suspended in 100 µL deionized water and administered to the column. Columns were rinsed with 600 µL deionized water before the derivatized glycans were eluted in 700 µL of nanopure water.

#### 2.7.3 Method Adaption for HILIC‐HPLC and Sample Preparation

Dionex Ultimate 3000 ultraviolet‐high‐performance liquid chromatography (UV‐HPLC)‐based glycan separation was achieved using HILIC chromatography on an XBridge Amide BEH Amide Column (130 Å, 3.5 µm, 2.1 mm, 150 mm) with a (130 Å, 3.5 µm, 2.1 mm, 5 mm) precolumn used according to the manufacturer's instructions.

A bicratic gradient of aqueous 100 mM ammonia formate (NH_4_Fo) buffer at pH 4.5 (Buffer A) and pure acetonitrile (ACN) (Buffer B) was used to separate the glycans. The total run time of the method was 104 min, consisting of a 1 min initial equilibration phase (22% B), followed by 79.26 min primary gradient in which B linearly increased to 44.1%. A subsequent wash gradient of 80% B was performed for 2.06 min and then held for 10.3 min, before flushing the system back to initial 22% B in 4.12 min and equilibration for 7.2 min. The temperature of the column was kept at 60°C and auto sampler temperature at 15°C. The injection volume was 18 µL. The detection was performed at 254 nm.

The previously evaporated samples were dissolved in 22 µL of conditioning mixture, consisting of ACN, 13 µL; MeOH, 1 µL; H_2_O, 3.16 and 4.84 µL of 100 µM NH_4_Fo buffer; and pH 4.5 directly before measurement. Glycan identification was performed by comparing normalized retention times (glucose units) with literature values [83] and the Ludger Human IgG N‐glycan Library (https://www.ludger.com/docs/tables/ludger‐human‐igg‐n‐glycan‐library‐table‐final.pdf).

For each pulsing regime, the specific galactosylation index (*I*_G_ [%]) was calculated as follows:

(1) $$I_{G}=\left(\frac{2\cdot \left[FA2G2\right]+\left[FA2G1\right]}{\left[FA2G2\right]+\left[FA2G1\right]+\left[FA2\right]}\right)\;\cdot 100$$

*I*_G_ serves as a measure of the relative abundance of galactose in glycans obtained under the given cultivation conditions.

### 2.8 Data Evaluation

LC‐MS/MS data were acquired using Agilent MassHunter acquisition software (B.06.00). Raw data processing and extraction were conducted using an Agilent Mass‐Hunter (B.06.00). HPLC data for mABs were acquired and processed using Chromeleon 7 software (Thermo Fisher). Data fitting for the determination of the sigmoid digitonin function was conducted using Matlab's fit function application (version: Matlab R2023b). All further data evaluations were conducted using Microsoft Excel. Except for the sigmoid plot, all the data were visualized using VEUSZ (version: 3.6.2). The data from this publication can be found under: https://doi.org/10.18419/DARUS‐4541.

## 3 Results

### 3.1 Subcellular Fractionation Separates Cells Into Compartments

**FIGURE 1:**
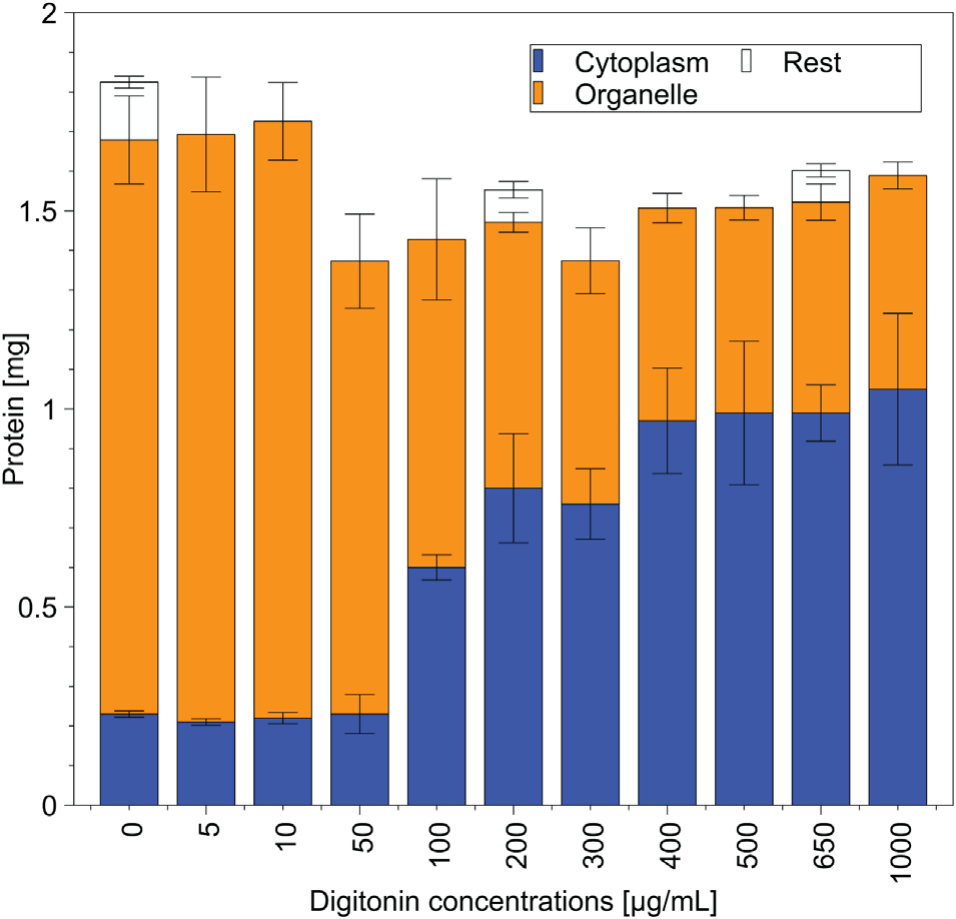
Protein mass in subcellular protein fractions. Protein mass (mg) over increasing digitonin concentration (µg/mL) in CP fractionation buffer. Fractions are CP (blue), GA (orange), or the remainder after fractionation (white). Error bars show standard deviation for mean values of *n* = 3. In rare cases, sample size had to be reduced.

Protein fractionation of CHO DP‐12 cells via digitonin separated the cellular contents in the CP (blue) and GA (orange) fractions (Figure 1). At low digitonin concentrations, almost all the proteins were observed in the organelle fraction. Only 0.22 ± 0.014 mg remained in the cytoplasm. Using digitonin levels > 50 µg/mL increased cytoplasmic protein content to approximately 0.97 ± 0.133 mg which was reached with 400 µg/mL. Extracts of pellets after fractionation showed low protein concentrations with < 0.15 ± 0.021 mg protein.

Western blot analysis confirmed the presence of the CP marker GAPDH in the CP fractions after treatment with more than 50 µg/mL digitonin (Figure S1). Notably, no organelle markers were detected (Figure S1). However, the latter was found in the GA fractions, irrespective of the digitonin concentration. The GA fraction contained only a negligible amount of the CP marker (Figure S1).

### 3.2 Subcellular Fractionation for Compartment‐Specific NSD Analysis

Encouraged by the successful fractionation, a methodology for compartment‐specific metabolome analysis was developed.

The workflow of this study builds on the preceding fractionation [33], considering adaptations to handle CHO DP‐12 and access the metabolites. The entire workflow consists of the following two parts: first, fractionation of cells into a CP fraction and an organelle fraction was achieved using digitonin, ending in thermal quenching of cellular metabolism to conserve metabolite integrity. Washing after sampling and removal of the first fraction ensured minimal cross‐contamination between fractions and prevented leftovers from the residual culture medium. In addition, to prevent degradation, the entire process was performed at pace and cooled at 4°C before quenching in liquid nitrogen (N_2_^lq^).

Second, metabolites were extracted using methanol and chloroform at −70°C [36] to ensure long‐term sample stability. Finally, the samples were concentrated to yield sufficient metabolite levels for HILIC‐QQQ‐MS/MS quantification.

#### 3.2.1 Lyophilization Achieves Optimal Sample Concentration by Preventing Analyte Loss

Due to the limited amount of analyte, sample concentration was assessed by comparing the processes of evaporation and lyophilization. The former was chosen as a practical and timely efficient method, whereas the latter represented a milder technology. These methods were compared with respect to their ability to conserve the native state of the analytes (Figure S4). Uridine diphosphate‐glucose/‐galactose (UDP‐Glc/Gal) degraded more during evaporation than during lyophilization (Figure S4). Increasing digitonin concentrations elevated UDP‐Glc/Gal levels from 1.39 ± 0.19 to 4.08 ± 0.69 fmol/cell in lyophilized samples, whereas titers in evaporated samples increased only marginally. The profiles of the other analytes did not show any diverging trends between concentration approaches.

Nucleotides also underwent degradation during evaporation, as detected by the presence of dephosphorylated species (Figure S3). During evaporation, di‐ and monophosphate species were the most prominent metabolites, whereas during lyophilization, a larger amount of naturally produced triphosphates was conserved (> 54%).

The energy charge (EC) values calculated based on the respective analyte quantifications were higher in the lyophilized samples (Table S6). On average, evaporation reached 63% of the EC values obtained from the lyophilized samples.

#### 3.2.2 Subcellular Fractionation Produces Compartment‐Specific NSD Fractions

**FIGURE 2:**
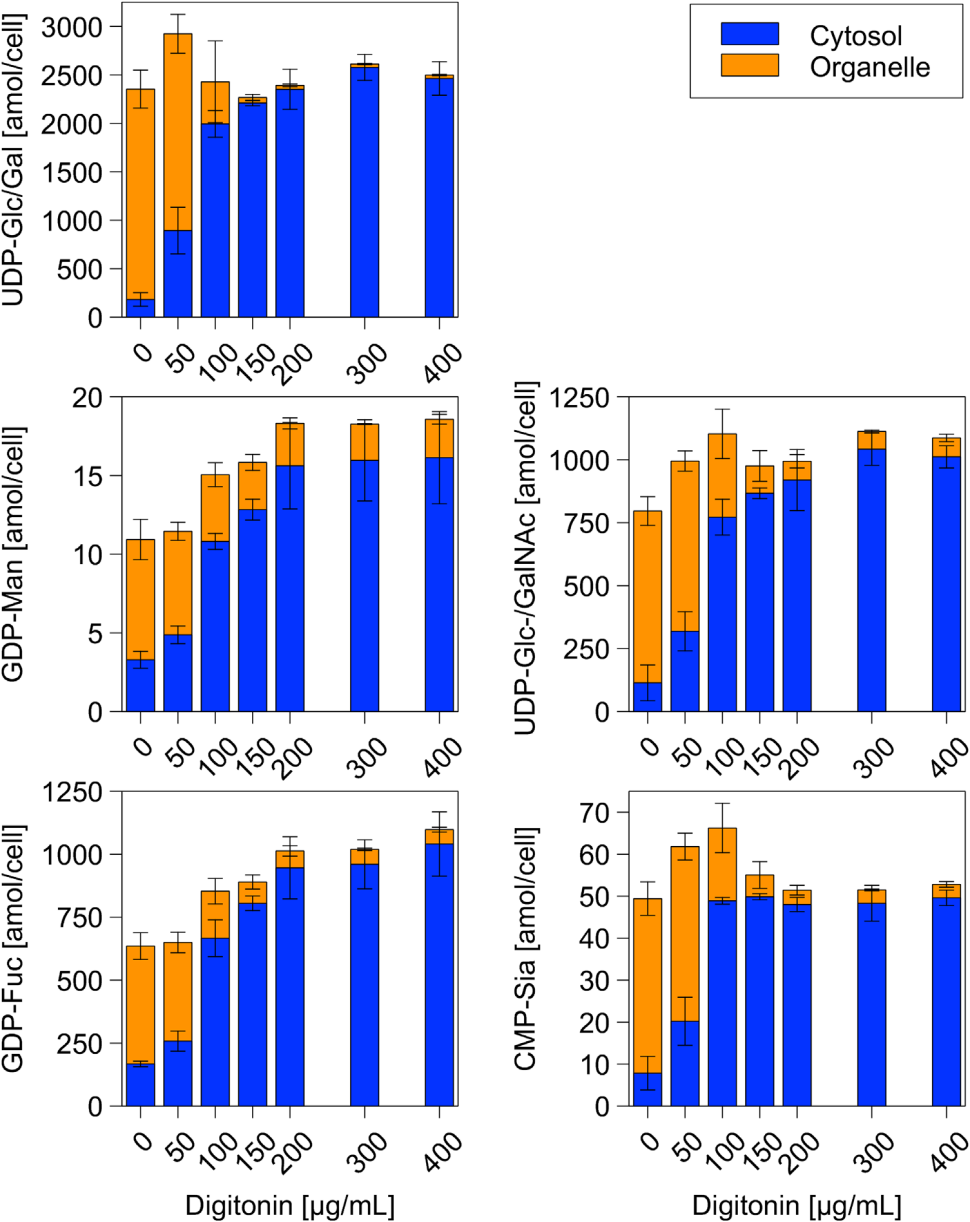
Metabolite concentration in subcellular metabolite fractions. Cellular metabolite concentration over increasing digitonin concentration in CP fractionation buffer. Fractions are CP fraction (blue) and GA (orange). Error bars show standard deviation for mean values of *n* = 3. In rare cases, sample size had to be reduced.

After successful lyophilization for sample concentration, subcellular metabolite fractionation was performed based on previously studied protein fractionation (Figure 2). Increasing additions of 50–200 µg/mL digitonin caused the sigmoidal rise of metabolite levels in the CP fraction (Figure 2). The phenomenon was observed for all analytes although individual levels range from 10.93 ± 1.82 amol/cell for guanosine diphosphate mannose (GDP‐Man) to 2923.58 ± 441.69 amol/cell for UDP‐Glc/Gal (Figure 2).

#### 3.2.3 Optimal Digitonin Concentration for Cell Fractionation Determined at 50 mg/mL

**FIGURE 3:**
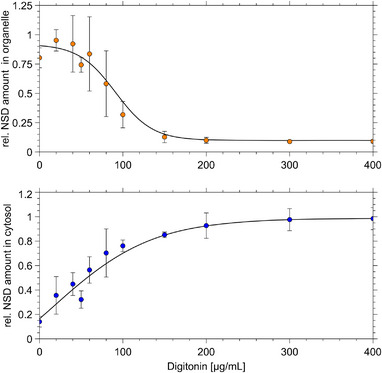
Sigmoid fit to metabolite concentrations. Averaged and normalized analyte concentrations are shown as organelle or CP fractions over increasing digitonin concentration in CP fractionation buffer. Averages over all measured metabolites and replicates (*n* = 3) were normalized to 1 at maximum cellular concentrations. Error bars show standard deviation for mean values of *n* = 3. *R* ^2^ = 0.996. In rare cases, the sample size had to be reduced.

Next, the optimum digitonin concentration for subcellular measurements was determined. To this end, the normalized average metabolite levels of all NSDs were plotted versus related digitonin titers and fitted to a sigmoid curve (Figure 3).

The fit yielded the following coefficients achieving a coefficient of determination (*R* ^2^) of 0.996 (Equation 2).

(2) $$y\;\left(x\right)=a+\frac{b}{1+e^{\left(\frac{x+c}{d}\right)}}\;$$

where *a* is 0.09805; *b*, 0.91998; *c*, 91.1277; *d*, 22.1166; *r* ^2^, 0.996; *x*, digitonin concentration in fractionation buffer (µg/mL); and *y*(*x*), average normalized amount metabolite (%).

Furthermore, literature values (Table S5) were used to estimate the compartment‐specific distribution of NSDs (Equations (3), (4), (5), (6), (7), (8), (9)).

Digitonin concentration was estimated applying the distribution in Equation (2).

(3) n=c·V

(4) $$r_{i}=\frac{n_{i}}{n_{Total}}$$

where, *r_i_* is rel. amount (%) of *i*; *n*,  amount (mol); *c*, concentration (mol/L); *V*, volume (L); *n*,   amount (mol)

(5) $$n_{\mathrm{Golgi}}=40\cdot 0.022=0.88\;$$

(6) $$n_{\mathrm{Cytoplasma}}=1\cdot 0.55=0.55\;\;$$

(7) nTotal=0.88+0.55=1.43∧=100%

(8) rGolgi=0,881,43≈0,62∧=62%

(9) rCytoplasma=0,551,43≈0,38∧=38%

Setting *y*(*x*) = 38% (0.38) in Equation (2) provides the needed digitonin concentration with 58 µg/mL. Therefore, 50 µg/mL was used in further experiments to minimize cross‐contamination from other organelles.

### 3.3 Subcellular Fractionation Allows Compartment‐Specific Quantification of Nutrient Pulse‐Induced Changes in NSD Supply

The digitonin concentration was used to perform metabolite fractionation tests. Compartment‐specific metabolite responses were monitored after the cells were exposed to different nutrient pulsing regimes intended to trigger NSD formation. All additions were performed in shaking flasks after 80.5 h.

(A) Addition of medium. (B) and (C) Media supplemented with manganese (II) chloride dihydrate (Mn), uridine, and sugar. Fructose was added to (B), whereas galactose was added to (C). Hence, (C) provides the entire precursor spectrum required to produce UDP‐galactose, providing Mn as a cofactor for glycosyl transferases. By contrast, (B) requires multiple transformation steps to produce an NSD. Accordingly, (A) served as a negative control to compensate for the changes in volume and effects based solely on the addition of the medium to the culture.

The different cultures showed high conformity with respect to cell growth, vitality, substrate consumption, and lactate formation (Figure S2). Similar cell diameters were observed. However, differences were observed with respect to cell count and viability. In particular, (A) showed significant losses in both, whereas the levels remained almost constant in (B) and (C) (Figure S2).

**FIGURE 4:**
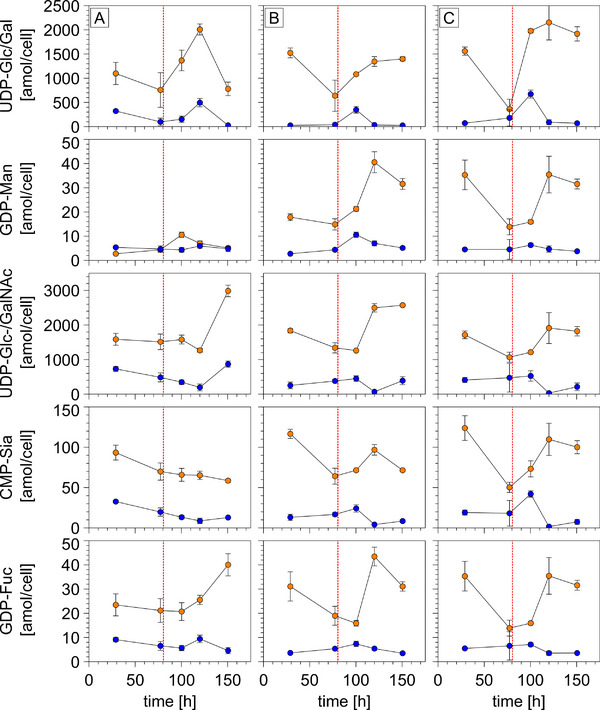
NSD levels in subcellular fractions over time. Concentrations in CP (blue) and GA (orange). Measured over 6 days with different nutrient pulsing strategies applied on Day 3: only medium (column left); Mn, uridine, and fructose dissolved in medium (column center); and Mn, uridine, and galactose dissolved in medium (column right). Pulsing time point indicated with red dotted line. Error bars show standard error of mean values for *n* = 3.

All pulsing strategies revealed metabolic responses in the CP and organelles with respect to the NSD levels (Figure 4). In the CP, only slight increases in NSD content were detected, which coincided with low metabolite levels compared to concentrations in the organelles. The concentration increases in the organelles were stronger in terms of amplitude and duration.

The amplitudes and slopes of the UDP‐Gal/Glc dynamics best illustrate the impact of the pulses (Figure 4). (A) resulted in a delayed noticeable increase in concentration in the CP as compared to conditions (B) and (C), where a pronounced increase in concentration in the CP was detected in the first sample taken after pulsing. However, in the GA, the increase under condition (B) was less steep than under condition (A). The UDP‐Glc/Gal concentration of condition (B) reached a lower maximum level of 1.4 ± 0.04 fmol/cell at 150.5 h, compared to condition (C), which reached 2.15 ± 0.04 fmol/cell at 120 h in the GA.

By analogy, GDP‐mannose concentrations in the CP and in the organelle remained in (A), whereas (B) and (C) induced similar rising metabolite levels but were delayed compared to UDP‐Glc/Gal.

Regarding uridine diphosphate N‐acetylglucosamine/‐galactosamine UDP‐GlcNAc/GalNAc, (A) triggered the latest response after 100 h in the organelles. In contrast, (B) and (C) induced somewhat earlier metabolite rises leading to 2.57 ± 0.03 and 1.82 ± 0.136 fmol/cell, respectively. The courses for guanosine diphosphate fucose (GDP‐Fuc) are similar, by trend. Regarding cytidine monophosphate N‐acetylneuraminate (CMP‐Sia), a high similarity exists for (B) and (C), whereas CMP‐Sia levels decreased consistently in the organelle for (A).

Interestingly, the concentrations of GDP‐Man, CMP‐Sia, and GDP‐Fuc were approximately 10‐ to 60‐fold lower than those of UDP‐Glc/Gal and UDP‐GlcNAc/GalNAc (Figure 4). Moreover, the dynamics of CP and organelles differ in frequency and amplitude.

### 3.4 Nutrient Pulsing Alters Glycopatterns of Produced Antibodies

The *I*_G_ indicated the degree of galactosylation at the end of cultivation. *I*_G_ was similar for pulsing strategies (A) and (B) (Table 1) whereas pulsing galactose (C) increased *I*_G_ to 88 ± 5%.

**TABLE 1:** Galactosylation index (*I*_G_) of cultivations under different nutrient pulsing strategies. Values were measured at the endpoint of cultivations. Values are means of *n* = 3 with standard error.

	A	B	C
**Galactosylation index (*I*_G_) (%)**	78 ± 12	75 ± 1	88 ± 8

In addition, pulsing (C) increased the amount of mono‐ and di‐galactosylated glycans compared to pulsing (B) (Figure S5), at absolute (top) and relative (bottom) levels. This effect was especially pronounced for di‐glycosylated FA2G2‐glycans. A significant increase in FA2G2‐glycan levels was observed after 122 h. Accordingly, FA2G2 showed the strongest increase in the relative proportion (Figure S5).

## 4 Discussion

### 4.1 Subcellular Fractionation Produces Highly Distinguishable Compartment Fractions for Proteins

Applying the protocol of Holden and Horton enabled the successful perforation of the plasma membrane [33]. Although the exact values for the protein measured and digitonin applied differed from those measured in HEK293 cells, the trajectories of the concentration curves depicted a highly conserved trend. This is further indicated by the specificity that could be shown using Western blotting for numerous organelles, including the GA [35, 41, 42].

At the start of the separation, the CP protein signal was likely biased by media residues and dead cells. After passing 50 µg/mL digitonin CP proteins permeate through the perforated membrane [43, 44, 45], reaching maximum permeability with > 300 µg/mL digitonin (Figure 1). The remaining proteins in the organelles are likely membrane‐bound proteins. Notably, digitonin preferentially perforates the CP membrane owing to its interaction with steroid structures [46, 47, 48]. Given that organelle membranes contain only 10%–46.7% cholesterol in CP membranes [49], the selective permeabilization of the CP membrane could be achieved.

Furthermore, Western blotting revealed the separation of marker proteins in the respective fractions (Figure S1), as shown for numerous organelles, including CP, ER, and GA [35, 41, 42]. In the CP fraction, the thin band for the marker GAPDH at 50 µg/mL digitonin agrees with the protein quantification tests. Both results indicated insufficient cell lysis at low levels of digitonin. In contrast, the presence of GAPDH residues in the Golgi apparatus likely indicates insufficient membrane perforation. This was because measures were taken to minimize undesired organelle lysis due to digitonin overexposure. This approach was favored to protect GA, as GA contains most of the target analytes, potentially leading to greater cross‐contamination. Further extraction of the samples yielded no significant protein content in the remaining pellets, suggesting that a representative fraction of protein was detected.

### 4.2 Subcellular Fractionation Can Be Adapted to Investigate NSDs

#### 4.2.1 NSD Degradation Is Prevented by Sufficient Cooling

This study aimed to advance the protein separation approach for compartment‐specific metabolite measurement. In addition to long‐term stable proteins, metabolic turnover is significantly faster, which may bias experimental readouts owing to improper sample procedures. As a proxy for estimating potentially biased sample processing times, all glycosylation reactions were lumped and considered as a single first‐order reaction. The relation between the reaction rate *k* and temperature *T* is given by the Arrhenius Equation (10) (*R* = universal gas constant [J/mol K], *E*_A_ = activation energy [J/mol], *T* = absolute temperature [K], *k*_0_ = reaction rate at reference conditions):

(10) $$k=k_{0}^{\left(-\frac{E_{A}}{R\cdot T}\right)}\;$$

It has previously been shown that the reaction rates are halved under ambient‐temperature conditions if the temperature is decreased by 10 K. This phenomenon is also known as the ‘‘Q10, quotient of 10 K rule’’ [50] and leads to the estimation of the reduced reaction rate *k*_cold_ compared to the reference (warm) reaction rate *k*_warm_ as follows:

(11) $$k_{\mathrm{cold}}=\frac{k_{\mathrm{warm}}}{\;2^{\left(\frac{\Delta T}{10}\right)}}\;$$

In other words, the time needed to produce the same amount of product with the reduced reaction rate *k*_cold_ is increased by the factor $\;2^{\left(\frac{\Delta T}{10}\right)}:$

(12) $$t_{\mathrm{cold}}=t_{\mathrm{warm}}\;\cdot \;2^{\left(\frac{\Delta T}{10}\right)}$$

Since the optimum growth temperature of CHO cells is at 37°C and fractionation was conducted at maximum 4°C, we calculate:

(13) ΔT=Tgrowth−Tfractionation=33∘C∧=33K

The processing time (*t*_Gly_) for antibody glycosylation within GA under optimal growth conditions has been reported to be 22 min [11, 51, 52]. Hence, applying Equation (12) yields:

(14) $$216.7\;\min\;=22\;\min\cdot 2^{\left(\frac{33}{10}\right)}$$

Given that glycosylation comprises seven major steps [51], the average time for a single catalytic step under cold conditions can be approximated as follows:

(15) $$\frac{216.7\;\min}{7\;\mathrm{steps}}\approx 31\;\min/\mathrm{step}$$

Hence, we concluded that triplicate sampling was fast enough to ensure sample integrity.

Remark: For simplification, we neglected the consumption of NSDs for the glycosylation of lipids because their contribution to the total NSD consumption is below 10% [53].

#### 4.2.2 Mild Conditions in Lyophilization Best Conserve Analytes During Concentration

A comparison of evaporation and lyophilization samples emphasizes the need to protect the targeted compounds by installing mild concentration conditions. The clearly measured EC values after lyophilization (Table S6) matched best with those of independent studies (Table S7), which highlights the strength of this approach (Figure S4).

#### 4.2.3 Subcellular Fractionation Is Transferable From Protein to NSDs

The sigmoidal trends of increasing fractionation with increasing digitonin exposure in the final stages indicate similar mechanisms for metabolite and protein separation. Notably, no marker metabolites for GA are currently known to serve as specific identifiers, which may further elucidate the fractionation mechanisms.

The significant concentration differences between the metabolite fractions at low digitonin exposure illustrate two phenomena: (1) large gradients exist for this metabolite in vivo and (2) low digitonin exposure does not bias the in vivo conditions.

Figure 2 shows that exposure to equal amounts of digitonin caused a stronger release of metabolites than proteins. Apparently, low amounts of digestion create small pores that enable the efflux of low‐molecular‐weight metabolites while retaining large proteins. This phenomenon was mirrored by the earlier and steeper ascent of the sigmoidal trend for the metabolites than for the proteins.

The isobaric NSD pairs with high structural similarity to UDP‐Glc/UDP‐Gal and UDP‐GlcNAc/UDP‐GalNAc could not be chromatographically separated and were, therefore, reported as mixed pools.

The current compartment‐specific analysis distinguished between the CP and organelle fractions. The latter comprises all the organelles in eukaryotic cells. However, we assigned the occurrence of NSDs solely to the organelle GA, the spot where protein N‐glycosylation is located and the location where the consumption of NSDs takes place. In addition, our study also considered NSD occurrence in the CP fraction, where NSD production and consumption occur. One example is the assembly of mannose‐ and glucose‐containing glycans. On the outer side of the endoplasmatic reticulum (ER) membrane, UDP‐Glc and GDP‐Man are converted to dolichol phosphate glucose (Dol‐P‐Glc) and dolichol phosphate mannose (Dol‐P‐Man) [54, 55]. The latter being the substrate for the initial glycan assembly that takes place inside the ER.

All subsequent steps that consume the NSD in the N‐glycosylation of proteins are performed within the GA [56]. Hence, the CP as the origin of NSDs and the GA as the processing compartment encompasses the predominant occurrence of NSDs.

#### 4.2.4 Operational Range of Digitonin Use

The feasible range of digitonin exposure was identified by deducing hints from independent studies [57, 58] and applying theoretical assumptions. The comparison of our measurements with the results of independent studies led to the conclusion that the suggested experimental protocol can be successfully applied.

The first piece of evidence was obtained by comparing the physiological energy states in our study with other research results (Tables S6 and S7). Further agreement was observed when the intracellular metabolite levels were compared with those of other approaches. Notably, no other compartment‐specific NSD data exist. However, the compartment‐specific data of this study are consistent with the total cell measurements in the literature [12, 59–61].

The initially assumed distribution ratio of NSDs in the GA versus CP of 40:1 overestimates the actual physiological conditions, with most observed ratios ranging from 2:1 to 20:1 (Figure 4). However, we observed a rapid pool size increase in the GA after NSD production began in the CP, as described by [11]. In summary, compartment‐specific pool sizes do not show the expected large distribution ratios nor are they stable. In addition, pool sizes and distribution ratios are unique for individual NSDs, which highlights the need to measure the NSD content in the GA and cytosol individually.

The measurements were further validated by comparison with independent studies. Specific CP and GA data lacking total cell concentrations were compared and revealed similarities within the same order of magnitude [12, 59–61].

This approach involves sequential sampling, selective permeabilization of the cytoplasmic membrane, subcellular fractionation, and thermal quenching of the obtained fractions. Subsequently, with the metabolic rates at rest, the fractions were permanently quenched via organic solvent extraction, followed by lyophilization, reconstitution, and the final targeted LC‐MS/MS approach. Until thermal quenching, sample processing was completed in less than 30 min, which prevented biased readouts (Equations (2), (3), (4), (5), (6)). Alternative approaches, such as differential centrifugation [28, 35] and density gradient centrifugation [62, 63] likely bias metabolite measurements because of their long processing times or higher temperatures. These procedures are well‐acknowledged for protein analysis but do not seem to be well‐suited for non‐biased metabolite analysis because of the necessity of nonspecific cellular lysis.

Several of these complications have recently been overcome using Golgi‐IP [30]. In contrast, our method can be performed without any genetic alteration in the analyzed cell line, thus increasing its applicability.

Notably, the current method can easily be scaled up regarding the number of processed samples at a time by further miniaturization, parallelization, and the use of automated robotics. Furthermore, its use may not be limited to CHO cells but may likely be transferrable to other eucaryotic cells, if selective perforation of the cytoplasmic membrane via digitonin could be equally achieved.

### 4.3 Nutrient Pulsing Induces Compartment‐Specific Responses in NSD Levels

Cells were fed different nutrient pulses to induce differential NSDs. In doing so, we checked whether the time series agreed with independent studies that previously investigated the impact of media on glycosylation patterns and precursors [12, 64–66].

In general, pulsing studies (A)–(C) caused statistically significant differences in the NSD concentration profiles.

**Puls (A)**: The delayed increase of cytoplasmatic UDP‐Glc/Gal levels and the earlier cell death (Figure S2) under condition (A), as compared to strategies (B) and (C), is consistent with the limited carbon availability under condition (A). This is in line with the concomitantly reduced availability of the cofactor Mn and the co‐substrate uridine. Consequently, most NSD concentrations reduced over cultivation time, especially in the CP [67]. The comparatively low and constant levels of GDP‐Man align with the understanding that no transporter exists in the ER or GA for this molecule [68]. In contrast, the strong and delayed increase of GDP‐Man in the GA under conditions (B) and (C) may be the result of transporter promiscuity, as described in other instances in the literature [24].

**Pulses (B) and (C)**: In contrast to strategy (A), the subcellular NSD responses after pulsing under conditions (B) and (C) commonly have concentrations that mostly return to pre‐pulse levels after the perturbations. After pulse (B), the subcellular NSD levels increased directly before returning to initial values. Pulse (C) shows a similar trend, however, with a more delayed NSD response. The pulse‐induced NSD spikes may have strongly impacted subcellular NSD transport [69]. del Val et al. previously outlined that intermittent NSD shortages inside the GA are unlikely to be due to transport limitations into the GA [11]. Instead, the transport mechanisms between the GA and CP appear to favor a strong influx into the GA. Notably, the CP NSD levels remained largely constant during pulsing, possibly as a cellular strategy to maintain CP homeostasis.

Deviations due to potential pulse dilution effects are unlikely. In this regard, the NSD influx is directed from the CP into a significantly smaller GA (4% of the CP volume [12, 70]). Due to the difference in size, any concentration increase by reverse transport from the GA into the CP would be negligible.

Remarkably, the interim CP concentrations of UDP‐Glc/GalNAc and CMP‐Sia were below the pre‐pulse levels before recovery. The depletion of UDP‐Glc/GalNAc may be explained by overregulation rooted in the substrate promiscuity of nucleotide sugar transporters (NSTs) for UDP‐Gal and UDP‐GlcNAc [71]. Given that CMP‐Sia is a derivative of UDP‐GlcNAc [72, 73, 74], the two dynamics are linked.

Increased NSD levels were frequently observed in the GA fractions. For instance, peak UDP‐Glc/Gal and GDP‐Man levels after pulse (B) mirror the utilization of fructose, as both NSDs are produced from Fru‐6P via a few enzymatic reactions. This effect was even more pronounced when galactose was consumed during pulse under condition (C); UDP‐Glc/Gal was converted directly from galactose‐1‐phosphate. The formation of UDP‐GlcNAc/GalNAc relies more on fructose than on galactose, which is why NSDs are more abundant under condition (B). Accordingly, the time delays and amplitudes of subsequent metabolite increases in the CP and GA correlate with the proximity of the pathway of the metabolite to the pulsed carbon source [72, 73, 74].

Increased NSD levels were frequently observed in the GA fractions. For instance, peak UDP‐Glc/Gal and GDP‐Man levels after pulse (B) mirror the utilization of fructose, as both NSDs are produced from Fru‐6P via a few enzymatic reactions. This effect was even more pronounced when galactose was consumed during pulse (C); UDP‐Glc/Gal was converted directly from galactose‐1‐phosphate. The formation of UDP‐GlcNAc/GalNAc relies more on fructose than on galactose, which is why NSDs are more abundant under condition (B). Accordingly, the time delays and amplitudes of subsequent metabolite increases in CP and GA correlate with the proximity of the pathway of the metabolite to the pulsed (C) source [72, 73, 74].

Independent pulsing experiments that aimed at influencing cellular glycosylation patterns have shown that the addition of selected substrates affects NSD patterns [64]. Furthermore, impacts on the glycosylation patterns of antibodies have been observed. The transport of NSDs into GA [69, 75] likely plays a key role, as indicated by our study.

Interestingly, affinity constants *K*_m_ of the GA transporters for NSDs are typically lower than the measured CP concentrations [58, 76–78]. Antiporter transport ensures NSD uptake into the GA against the concentration gradient [79, 80]. Cells prevent the accumulation of ‘‘metabolically expensive’’ NSDs in the GA by controlling counterion availability [81]. Inside the GA, concentrations of NSDs are typically close to the *K*_m_ values of respective glycosyltransferases, that is, glycosylation is initially metabolically controlled rather than limited by the availability of active glycosyltransferases.

The findings of this study are overall in line with other independent findings. This holds true for the relative proportion of NSDs in GA and CP [30] as well as for measured NSD levels [12, 59–61], which highlights the quality of the approach.

### 4.4 Observed NSD Supply Impacts Antibody Glycosylation

The detected glycan pattern was consistent with the results of other studies [82]. In addition, the rising *I*_G_ after nutrient pulsing was consistent with other findings [12, 65, 66]. Furthermore, the increasing *I*_G_ mirrors the detected increase in UDP‐Glc/Gal production, which complements our understanding of glycosylation mechanisms. This holds also true for the availability of the other NSDs and their impact on the *I*_G_, as shown in this approach (Figure S5).

## 5 Conclusion

A novel analytical workflow was developed by adapting well‐established fractionation techniques and combining them with state‐of‐the‐art metabolomics sampling and sample processing procedures. Lyophilization was identified as the optimal technique for sample concentration due to its ability to preserve targeted analytes under mild conditions. The necessary amount of digitonin for compartment‐specific cell fractionation was determined to be 50 µg/mL. The workflow was finalized by sophisticated quantitative LC‐MS/MS measurements. This approach enables compartment‐specific isolation and analysis of NSDs, providing quantitative insights into the subcellular transport mechanisms of NSDs as part of the eukaryotic protein glycosylation process in CHO cells.

The presented method was successfully employed to depict metabolic changes in the NSD supply based on nutrient pulsing strategies. These subcellular changes were matched by extracellular glycosylation patterns on produced antibodies, showcasing the methods great fit for its purpose. The findings demonstrate that NSD supply into the GA is an important factor in regulating N‐glycosylation.

Evidently, NSD transport into the GA is characterized by a strong influx of NSDs into the GA. As we demonstrated, NSD concentrations inside the GA are, in most cases, very close to transporters’ *K*_M_ values. In contrast, CP concentrations are far lower, allowing for transport to be controlled metabolically from within the GA.

Other forms of carbohydrate‐based protein modifications, such as glycation or O‐glycosylation, were not investigated in this study.

Future studies should focus on a better understanding of the subcellular metabolic response and NSD transport dynamics resulting from alternative cultivation strategies and targeted external stimuli. The presented method has the potential to facilitate compliance with the strict regulatory assessments in place today and improve modern control strategies for glycan patterns during antibody production processes.

## Author Contributions

 **Niklas Regett**: Conceptualization (Equal), Data curation (Lead), Formal analysis (Lead), Investigation (Lead), Methodology (Lead), Visualization (Lead), Writing ‐ original draft (Lead), **Marcel Dieterle**: Data curation (Supporting), Investigation (Supporting), Visualization (Supporting), **Fleur Peters**: Data curation (Supporting), Investigation (Supporting), Visualization (Supporting), **Max Deuring**: Data curation (Supporting), Investigation (Supporting), Visualization (Supporting), **Kaja Stegmaier**: Data curation (Supporting), Investigation (Supporting), Visualization (Supporting), **Attila Teleki**: Conceptualization (Supporting), Methodology (Supporting), Project administration (Supporting), Supervision (Equal), Writing ‐ review & editing (Supporting), **Ralf Takors**: Conceptualisation (Equal), Funding acquisition (Lead), Project administration (Lead), Resources (Lead), Supervision (Equal), Writing ‐ review & editing (Equal).

## Ethics Statement

No animals or human subjects were investigated in this study.

## Conflicts of Interest

The authors declare no conflicts of interest.

## Supporting information



Supporting Information

## Data Availability

The datasets from this study are available at https://doi.org/10.18419/darus‐4541. Further information can be requested from the corresponding author, Ralf Takors (ralf.takors@ibvt.uni‐stuttgart.de). Data includes nucleotide sugar donor (NSD) concentrations, antibody glycan distributions, and related metabolomic and protein data, obtained via subcellular fractionation, exhaustive sample extraction, and LC‐QQQ MS/MS techniques.

## References

[1] P. Krzyszczyk , A. Acevedo , E. J. Davidoff , et al., “The Growing Role of Precision and Personalized Medicine for Cancer Treatment,” Technology (Singapore World Science) 06, no. 3‐4 (2019): 79–100, 10.1142/S2339547818300020. PMC6352312 30713991

[2] N. M. Moussa‐Pacha , S. M. Abdin , H. A. Omar , H. Alniss , and T. H. Al‐Tel , “BACE1 Inhibitors: Current Status and Future Directions in Treating Alzheimer's Disease,” Medicinal Research Reviews 40, no. 1 (2020): 339–384, 10.1002/MED.21622. 31347728

[3] M. Yasunaga , “Antibody Therapeutics and Immunoregulation in Cancer and Autoimmune Disease,” Seminars in Cancer Biology 64 (2020): 1–12, 10.1016/J.SEMCANCER.2019.06.001. 31181267

[4] J. M. Reichert , C. J. Rosensweig , L. B. Faden , and M. C. Dewitz , “Monoclonal Antibody Successes in the Clinic,” Nature Biotechnology 23, no. 9 (2005): 1073–1078, 10.1038/nbt0905-1073. 16151394

[5] R. M. Lu , Y. C. Hwang , I. J. Liu , et al., “Development of Therapeutic Antibodies for the Treatment of Diseases,” Journal of Biomedical Science 27, no. 1 (2020): 1–30, 10.1186/S12929-019-0592-Z. 31894001 PMC6939334

[6] J. A. Oates , A. J. J. Wood , and J. M. Dwyer , “Manipulating the Immune System With Immune Globulin,” New England Journal of Medicine 326, no. 2 (1992): 107–116, 10.1056/NEJM199201093260206. 1727218

[7] F. Bray , M. Laversanne , H. Sung , et al., “Global Cancer Statistics 2022: GLOBOCAN Estimates of Incidence and Mortality Worldwide for 36 Cancers in 185 Countries,” CA: A Cancer Journal for Clinicians 74, no. 3 (2024): 229–263, 10.3322/CAAC.21834. 38572751

[8] E. Moorkens , A. G. Vulto , and I. Huys , “An Overview of Patents on Therapeutic Monoclonal Antibodies in Europe: Are They a Hurdle to Biosimilar Market Entry?,” Monoclonal Antibodies 12, no. 1 (2020), 1743517, 10.1080/19420862.2020.1743517. 32306833 PMC7188399

[9] S. Hutter , T. K. Villiger , D. Brühlmann , et al., “Glycosylation Flux Analysis Reveals Dynamic Changes of Intracellular Glycosylation Flux Distribution in Chinese Hamster Ovary Fed‐Batch Cultures,” Metabolic Engineering 43 (2017): 9–20, 10.1016/J.YMBEN.2017.07.005. 28754360

[10] P. M. Jedrzejewski , I. J. del Val , A. Constantinou , et al., “Towards Controlling the Glycoform: A Model Framework Linking Extracellular Metabolites to Antibody Glycosylation,” International Journal of Molecular Sciences 15, no. 3 (2014): 4492–4522, 10.3390/IJMS15034492. 24637934 PMC3975410

[11] I. J. del Val , J. M. Nagy , and C. Kontoravdi , “A Dynamic Mathematical Model for Monoclonal Antibody N‐linked Glycosylation and Nucleotide Sugar Donor Transport Within a Maturing Golgi Apparatus,” Biotechnology Progress 27, no. 6 (2011): 1730–1743, 10.1002/BTPR.688. 21956887

[12] P. Kotidis , P. Jedrzejewski , S. N. Sou , et al., “Model‐Based Optimization of AntiBody Galactosylation in CHO Cell Culture,” Biotechnology and Bioengineering 116, no. 7 (2019): 1612–1626, 10.1002/BIT.26960. 30802295

[13] S. Sha , Z. Huang , C. D. Agarabi , S. C. Lute , K. A. Brorson , and S. Yoon , “Prediction of N‐Linked Glycoform Profiles of Monoclonal Antibody With Extracellular Metabolites and Two‐Step Intracellular Models,” Processes 7, no. 4 (2019): 227, 10.3390/PR7040227.

[14] J. M. Hayes , A. Frostell , E. F. Cosgrave , et al., “Fc Gamma Receptor Glycosylation Modulates the Binding of IgG Glycoforms: A Requirement for Stable Antibody Interactions,” Journal of Proteome Research 13, no. 12 (2014): 5471–5485, 10.1021/PR500414Q. 25345863

[15] M. Movahedin , T. M. Brooks , N. T. Supekar , N. Gokanapudi , G. J. Boons , and C. L. Brooks , “Glycosylation of MUC1 Influences the Binding of a Therapeutic Antibody by Altering the Conformational Equilibrium of the Antigen,” Glycobiology 27, no. 7 (2017): 677–687, 10.1093/GLYCOB/CWW131. 28025250 PMC5881634

[16] S. W. Coates , T. Gurney , L. W. Sommerss , M. Yehs , and C. B. Hirschbergfe , “Subcellular Localization of Sugar Nucleotide Synthetases*,” Journal of Biological Chemistry 255, no. 19 (1980): 9225–9229, 10.1016/S0021-9258(19)70550-X. 6251080

[17] A. K. Münster , M. Eckhardt , B. Potvin , M. Mühlenhoff , P. Stanley , and R. Gerardy‐Schahn , “Mammalian Cytidine 5′‐Monophosphate N‐Acetylneuraminic Acid Synthetase: A Nuclear Protein With Evolutionarily Conserved Structural Motifs,” Proceedings of the National Academy of Sciences of the United States of America 95, no. 16 (1998): 9140–9145, 10.1073/PNAS.95.16.9140. 9689047 PMC21305

[18] G. Palade , “Intracellular Aspects of the Process of Protein Synthesis,” Science (New York, N.Y.) 189, no. 4200 (1975): 347–358, 10.1126/SCIENCE.1096303/ASSET/3AA88626-66C9-4CD7-A062-B134E9D52369/ASSETS/SCIENCE.1096303.FP.PNG. 1096303

[19] M. Butler , “Optimisation of the Cellular Metabolism of Glycosylation for Recombinant Proteins Produced by Mammalian Cell Systems,” Cytotechnology 50, no. 1 (2006): 57–76, 10.1007/S10616-005-4537-X. 19003071 PMC3476007

[20] P. Hossler , S. F. Khattak , and Z. J. Li , “Optimal and Consistent Protein Glycosylation in Mammalian Cell Culture,” Glycobiology 19, no. 9 (2009): 936–949, 10.1093/GLYCOB/CWP079. 19494347

[21] J. P. Kunkel , D. C. H. Jan , M. Butler , and J. C. Jamieson , “Comparisons of the Glycosylation of a Monoclonal Antibody Produced Under Nominally Identical Cell Culture Conditions in Two Different Bioreactors,” Biotechnology Progress 16, no. 3 (2000): 462–470, 10.1021/BP000026. 10835250

[22] J. Geigert , “Regulatory Pathways Impacting Biopharmaceuticals,” The Challenge of CMC Regulatory Compliance for Biopharmaceuticals (2023): 31–55, 10.1007/978-3-031-31909-9_2.

[23] A. F. Scheper , J. Schofield , R. Bohara , T. Ritter , and A. Pandit , “Understanding Glycosylation: Regulation Through the Metabolic Flux of Precursor Pathways,” Biotechnology Advances 67 (2023): 108184, 10.1016/J.BIOTECHADV.2023.108184. 37290585

[24] D. Maszczak‐Seneczko , M. Wiktor , E. Skurska , W. Wiertelak , and M. Olczak , “Delivery of Nucleotide Sugars to the Mammalian Golgi: A Very Well (un)Explained Story,” International Journal of Molecular Sciences 23, no. 15 (2022): 8648, 10.3390/IJMS23158648. 35955785 PMC9368800

[25] B. Teusink , J. Passarge , C. A. Reijenga , et al., “Can Yeast Glycolysis be Understood Terms of Vitro Kinetics of the Constituent Enzymes? Testing Biochemistry,” European Journal of Biochemistry 267, no. 17 (2000): 5313–5329, 10.1046/J.1432-1327.2000.01527.X. 10951190

[26] K. Van Eunen , J. A. L. Kiewiet , H. V. Westerhoff , and B. M. Bakker , “Testing Biochemistry Revisited: How In Vivo Metabolism Can Be Understood From In Vitro Enzyme Kinetics,” PLoS Computational Biology 8, no. 4 (2012): 1002483, 10.1371/journal.pcbi.1002483. PMC3343101 22570597

[27] W. E. Balch , W. G. Dunphy , W. A. Braell , and J. E. Rothman , “Reconstitution of the Transport of Protein Between Successive Compartments of the Golgi Measured by the Coupled Incorporation of N‐acetylglucosamine,” Cell 39, no. 2 pt. 1 (1984): 405–416, 10.1016/0092-8674(84)90019-9. 6498939

[28] J. H. Ehrenreich , J. J. M. Bergeron , P. Siekevitz , and G. E. Palade , “Golgi Fractions Prepared From Rat Liver Homogenates I. Isolation Procedure and Morphological Characterization,” Journal of Cell Biology 59, no. 1 (1973): 45–72, 10.1083/JCB.59.1.45. 4356571 PMC2110914

[29] S. Qin , Y. Zhang , Y. Tian , F. Xu , and P. Zhang , “Subcellular Metabolomics: Isolation, Measurement, and Applications,” Journal of Pharmaceutical and Biomedical Analysis 210 (2022): 114557, 10.1016/J.JPBA.2021.114557. 34979492

[30] R. Fasimoye , W. Dong , R. S. Nirujogi , et al., “Golgi‐IP, A Tool for Multimodal Analysis of Golgi Molecular Content,” Proceedings of the National Academy of Sciences of the United States of America 120, no. 20 (2023), e2219953120, 10.1073/PNAS.2219953120. 37155866 PMC10193996

[31] L. Junghans , A. Teleki , A. W. Wijaya , M. Becker , M. Schweikert , and R. Takors , “From Nutritional Wealth to Autophagy: In Vivo Metabolic Dynamics in the Cytosol, Mitochondrion and Shuttles of IgG Producing CHO Cells Compartment‐Specific Metabolomics CHO Recombinant Protein Production Cell Line Engineering Targets,” Metabolic Engineering 54 (2019): 145–159, 10.1016/j.ymben.2019.02.005. 30930288

[32] J. Pfizenmaier , J. C. Matuszczyk , and R. Takors , “Changes in Intracellular ATP‐Content of CHO Cells as Response to Hyperosmolality,” Biotechnology Progress 31, no. 5 (2015): 1212–1216, 10.1002/BTPR.2143. 26146937

[33] P. Holden and W. Horton , “Crude Subcellular Fractionation of Cultured Mammalian Cell Lines,” BMC Research Notes 2 (2009): 243, 10.1186/1756-0500-2-243. 20003239 PMC2802353

[34] U. K. Laemmli , “Cleavage of Structural Proteins During the Assembly of the Head of Bacteriophage T4,” Nature 227, no. 5259 (1970): 680–685, 10.1038/227680A0. 5432063

[35] S. Pérez‐Rodriguez , M. de Jesús Ramírez‐Lira , T. Wulff , et al., “Enrichment of Microsomes From Chinese Hamster Ovary Cells by Subcellular Fractionation for Its Use in Proteomic Analysis,” PLoS ONE 15, no. 8 (2020): e0237930, 10.1371/JOURNAL.PONE.0237930. 32841274 PMC7447005

[36] E. G. Bligh and W. J. Dyer , “A Rapid Method of Total Lipid Extraction and Purification,” Canadian Journal of Biochemistry and Physiology 37, no. 8 (1959): 911–917, 10.1139/O59-099. 13671378

[37] A. Teleki , A. Sánchez‐Kopper , and R. Takors , “Alkaline Conditions in Hydrophilic Interaction Liquid Chromatography for Intracellular Metabolite Quantification Using Tandem Mass Spectrometry,” Analytical Biochemistry 475 (2015): 4–13, 10.1016/j.ab.2015.01.002. 25600449

[38] A. Feith , A. Teleki , M. Graf , L. Favilli , and R. Takors , “Hilic‐Enabled13c Metabolomics Strategies: Comparing Quantitative Precision and Spectral Accuracy of Qtof High‐ and Qqq Low‐resolution Mass Spectrometry,” Metabolites 9, no. 4 (2019): 63, 10.3390/metabo9040063. 30986989 PMC6523712

[39] J. C. Bigge , T. P. Patel , J. A. Bruce , P. N. Goulding , S. M. Charles , and R. B. Parekh , “Nonselective and Efficient Fluorescent Labeling of Glycans Using 2‐Amino Benzamide and Anthranilic Acid,” Analytical Biochemistry 230, no. 2 (1995): 229–238, 10.1006/ABIO.1995.1468. 7503412

[40] M. Melmer , T. Stangler , M. Schiefermeier , et al., “HILIC Analysis of Fluorescence‐Labeled N‐Glycans From Recombinant Biopharmaceuticals,” Analytical and Bioanalytical Chemistry 398, no. 2 (2010): 905–914, 10.1007/S00216-010-3988-X/FIGURES/7. 20640408

[41] H. Plutner , H. W. Davidson , J. Saraste , and W. E. Balch , “Morphological Analysis of Protein Transport From the ER to Golgi Membranes in Digitonin‐Permeabilized Cells: Role of the P58 Containing Compartment,” Journal of Cell Biology 119, no. 5 (1992): 1097–1116, 10.1083/JCB.119.5.1097. 1447290 PMC2289727

[42] M. Wibo , D. Thines‐Sempoux , A. Amar‐Costesec , H. Beaufay , and D. Godelaine , “Analytical Study of Microsomes and Isolated Subcellular Membranes From Rat Liver VIII. Subfractionation of Preparations Enriched With Plasma Membranes, Outer Mitochondrial Membranes, or Golgi Complex Membranes,” Journal of Cell Biology 89, no. 3 (1981): 456–474, 10.1083/JCB.89.3.456. 7251662 PMC2111792

[43] A. H. Fischer , K. A. Jacobson , J. Rose , and R. Zeller , “Fixation and Permeabilization of Cells and Tissues,” Cold Spring Harbor Protocols 2008, no. 5 (2008): pdb top36, 10.1101/PDB.TOP36. 21356837

[44] M. E. Stearns and R. L. Ochs , “A Functional In Vitro Model for Studies of Intracellular Motility in Digitonin‐Permeabilized Erythrophores,” Journal of Cell Biology 94, no. 3 (1982): 727–739, 10.1083/JCB.94.3.727. 6215414 PMC2112209

[45] P. F. Zuurendonk and J. M. Tager , “Rapid Separation of Particulate Components and Soluble Cytoplasm of Isolated Rat‐Liver Cells,” Biochimica Et Biophysica Acta (BBA)—Bioenergetics 333, no. 2 (1974): 393–399, 10.1016/0005-2728(74)90022-X. 19400050

[46] N. Frenkel , A. Makky , I. R. Sudji , M. Wink , and M. Tanaka , “Mechanistic Investigation of Interactions Between Steroidal Saponin Digitonin and Cell Membrane Models,” Journal of Physical Chemistry B 118, no. 50 (2014): 14632–14639, 10.1021/JP5074939. 25412206

[47] R. Malabed , S. Hanashima , M. Murata , and K. Sakurai , “Sterol‐Recognition Ability and Membrane‐Disrupting Activity of Ornithogalum Saponin OSW‐1 and Usual 3‐O‐glycosyl Saponins,” Biochimica Et Biophysica Acta (BBA)—Biomembranes 1859, no. 12 (2017): 2516–2525, 10.1016/J.BBAMEM.2017.09.019. 28947142

[48] M. Orczyk , K. Wojciechowski , and G. Brezesinski , “The Influence of Steroidal and Triterpenoid Saponins on Monolayer Models of the Outer Leaflets of Human Erythrocytes, E. coli and S. cerevisiae Cell Membranes,” Journal of Colloid & Interface Science 563 (2020): 207–217, 10.1016/J.JCIS.2019.12.014. 31874308

[49] J. D. Lambris and R. Paoletti , “ *Lipids in Protein Misfolding* ,” in *Advances in Experimental Medicine and Biology* , vol. 855 (Springer International Publishing, 2015), 10.1007/978-3-319-17344-3.

[50] I. A. Leenson , “Old Rule of Thumb and the Arrhenius Equation,” Journal of Chemical Education 76, no. 10 (1999): 1459–1460, 10.1021/ED076P1459.

[51] I. Arigoni‐Affolter , E. Scibona , C. W. Lin , et al., “Mechanistic Reconstruction of Glycoprotein Secretion Through Monitoring of Intracellular N‐Glycan Processing,” Science Advances 5, no. 11 (2019): eaax8930, 10.1126/sciadv.aax8930. 31807707 PMC6881162

[52] K. Hirschberg and J. Lippincott‐Schwartz , “Secretory Pathway Kinetics and In Vivo Analysis of Protein Traffic From the Golgi Complex to the Cell Surface,” The FASEB Journal 13, no. 9002 (1999): S251–S256, 10.1096/FASEBJ.13.9002.S251. 10619138

[53] I. J. Del Val , K. M. Polizzi , and C. Kontoravdi , “A Theoretical Estimate for Nucleotide Sugar Demand Towards Chinese Hamster Ovary Cellular Glycosylation,” Scientific Reports 6, no. 1 (2016): 1–15, 10.1038/srep28547. 27345611 PMC4921913

[54] M. Aebi , “N‐Linked Protein Glycosylation in the ER,” Biochimica et Biophysica Acta (BBA) ‐ Molecular Cell Research 1833, no. 11 (2013): 2430–2437, 10.1016/J.BBAMCR.2013.04.001. 23583305

[55] J. Breitling and M. Aebi , “N‐Linked Protein Glycosylation in the Endoplasmic Reticulum,” Cold Spring Harbor Perspectives in Biology 5, no. 8 (2013): a013359, 10.1101/CSHPERSPECT.A013359. 23751184 PMC3721281

[56] P. Stanley , “Golgi Glycosylation,” Cold Spring Harbor Perspectives in Biology 3, no. 4 (2011): a005199, 10.1101/CSHPERSPECT.A005199. 21441588 PMC3062213

[57] M. Perez and C. B. Hirschberg , “Translocation of UDP‐N‐Acetylglucosamine Into Vesicles Derived From Rat Liver Rough Endoplasmic Reticulum and Golgi Apparatus,” Journal of Biological Chemistry 260, no. 8 (1985): 4671–4678, 10.1016/s0021-9258(18)89122-0. 3988731

[58] B. C. Waldman and G. Rudnick , “UDP‐GlcNAc Transport Across the Golgi Membrane: Electroneutral Exchange for Dianionic UMP,” Biochemistry 29, no. 1 (1990): 44–52, 10.1021/BI00453A006/ASSET/BI00453A006.FP.PNG_V03. 2322548

[59] C. F. Shek , P. Kotidis , and M. Betenbaugh , “Mechanistic and Data‐Driven Modeling of Protein Glycosylation,” Current Opinion in Chemical Engineering 32 (2021): 100690, 10.1016/J.COCHE.2021.100690.

[60] Y. Fan , I. Jimenez Del Val , C. Müller , et al., “Amino Acid and Glucose Metabolism in Fed‐Batch CHO Cell Culture Affects Antibody Production and Glycosylation,” Biotechnology and Bioengineering 112, no. 3 (2015): 521–535, 10.1002/BIT.25450. 25220616

[61] S. N. Sou , P. M. Jedrzejewski , K. Lee , C. Sellick , K. M. Polizzi , and C. Kontoravdi , “Model‐Based Investigation of Intracellular Processes Determining Antibody Fc‐glycosylation Under Mild Hypothermia,” Biotechnology and Bioengineering 114, no. 7 (2017): 1570–1582, 10.1002/BIT.26225. 27869292 PMC5485029

[62] J. M. Graham , “Isolation of Golgi Membranes From Tissues and Cells by Differential and Density Gradient Centrifugation,” Current Protocols in Cell Biology 10, no. 1 (2001): 3.9.1–3.9.24, 10.1002/0471143030.CB0309S10. 18228361

[63] X. Li and M. Donowitz , “Fractionation of SubCellular Membrane Vesicles of Epithelial and Nonepithelial Cells by optiPrep^TM^ Density Gradient Ultracentrifugation,” Methods in Molecular Biology 440 (2008): 97–110, 10.1007/978-1-59745-178-9_8/FIGURES/4_8. 18369940

[64] E. J. M. Blondeel , K. Braasch , T. McGill , et al., “Tuning a MAb Glycan Profile in Cell Culture: Supplementing N‐acetylglucosamine to Favour G0 Glycans Without Compromising Productivity and Cell Growth,” Journal of Biotechnology 214 (2015): 105–112, 10.1016/j.jbiotec.2015.09.014. 26387447

[65] R. K. Grainger and D. C. James , “CHO Cell Line Specific Prediction and Control of Recombinant Monoclonal Antibody N‐Glycosylation,” Biotechnology and Bioengineering 110, no. 11 (2013): 2970–2983, 10.1002/BIT.24959. 23737295

[66] J. Liu , J. Wang , L. Fan , et al., “Galactose Supplementation Enhance Sialylation of Recombinant Fc‐Fusion Protein in CHO Cell: An Insight Into the Role of Galactosylation in Sialylation,” World Journal of Microbiology and Biotechnology 31, no. 7 (2015): 1147–1156, 10.1007/S11274-015-1864-8/METRICS. 25931375

[67] C. Villacrés , V. S. Tayi , E. Lattová , H. Perreault , and M. Butler , “Low Glucose Depletes Glycan Precursors, Reduces Site Occupancy and Galactosylation of a Monoclonal Antibody in CHO Cell Culture,” Biotechnology Journal 10, no. 7 (2015): 1051–1066, 10.1002/BIOT.201400662. 26058832

[68] C. B. Hirschberg , “Transport of Nucleotide Sugars, Nucleotide Sulfate and ATP Into the Lumen of the Golgi Apparatus,” Golgi Apparatus (1997): 163–178, 10.1007/978-3-0348-8876-9_5.

[69] W. R. Pels Rijcken , B. Overdijk , D. H. Van Den Eijnden , and W. Ferwerda , “The Effect of Increasing Nucleotide‐Sugar Concentrations on the Incorporation of Sugars Into Glycoconjugates in Rat Hepatocytes,” Biochemical Journal 305 (1995): 865–870. 7848287 10.1042/bj3050865 PMC1136339

[70] B. Alberts , A. Johnson , J. Lewis , M. Raff , K. Roberts , and P. Walter , “Molecular Biology of the Cell, 5th Edition by B. Alberts, A. Johnson, J. Lewis, M. Raff, K. Roberts, and P. Walter,” Biochemistry and Molecular Biology Education 36, no. 4 (2008): 317–318, 10.1002/BMB.20192.

[71] M. Olczak , D. Maszczak‐Seneczko , P. Sosicka , P. Jakimowicz , and T. Olczak , “UDP‐Gal/UDP‐GlcNAc Chimeric Transporter Complements Mutation Defect in Mammalian Cells Deficient in UDP‐Gal Transporter,” Biochemical and Biophysical Research Communications 434, no. 3 (2013): 473–478, 10.1016/J.BBRC.2013.03.098. 23583405

[72] M. Kanehisa , “Toward Understanding the Origin and Evolution of Cellular Organisms,” Protein Science 28, no. 11 (2019): 1947–1951, 10.1002/PRO.3715. 31441146 PMC6798127

[73] M. Kanehisa , M. Furumichi , Y. Sato , M. Kawashima , and M. Ishiguro‐Watanabe , “KEGG for Taxonomy‐Based Analysis of Pathways and Genomes,” Nucleic Acids Research 51, no. D1 (2023): D587–D592, 10.1093/NAR/GKAC963. 36300620 PMC9825424

[74] M. Kanehisa and S. Goto , “KEGG: Kyoto Encyclopedia of Genes and Genomes,” Nucleic Acids Research 28, no. 1 (2000): 27–30, 10.1093/NAR/28.1.27. 10592173 PMC102409

[75] D. J. Carey , L. W. Sommers , and C. B. Hirschberg , “CMP‐N‐Acetylneuraminic Acid: Isolation From and Penetration Into Mouse Liver Microsomes,” Cell 19, no. 3 (1980): 597–605, 10.1016/S0092-8674(80)80036-5. 7363326

[76] A. Lepers , L. Shaw , R. Cacan , R. Schauer , J. Montreuil , and A. Verbert , “Transport of CMP‐N‐Glycoloylneuraminic Acid Into Mouse Liver Golgi Vesicles,” Federation of European Biochemical Societies Letters 250, no. 2 (1989): 245–250, 10.1016/0014-5793(89)80731-8. 2753135

[77] M. E. Milla , C. A. Clairmont , and C. B. Hirschberg , “Reconstitution Into Proteoliposomes and Partial Purification of the Golgi Apparatus Membrane UDP‐Galactose, UDP‐Xylose, and UDP‐glucuronic Acid Transport Activities,” Journal of Biological Chemistry 267, no. 1 (1992): 103–107, 10.1016/S0021-9258(18)48465-7. 1730575

[78] L. W. Sommers and C. B. Hirschberg , “Transport of Sugar Nucleotides Into Rat Liver Golgi. A New Golgi Marker Activity,” Journal of Biological Chemistry 257, no. 18 (1982): 10811–10817, 10.1016/s0021-9258(18)33897-3. 7050120

[79] C. B. Hirschberg , “Golgi Nucleotide Sugar Transport and Leukocyte Adhesion Deficiency II,” Journal of Clinical Investigation 108, no. 1 (2001): 3–6, 10.1172/JCI13480. 11435449 PMC209350

[80] C. B. Hirschberg , P. W. Robbins , and C. Abeijon , “Transporters of Nucleotide Sugars, ATP, and Nucleotide Sulfate in the Endoplasmic Reticulum and Golgi Apparatus,” Annual Review of Biochemistry 67 (1998): 49–69, 10.1146/ANNUREV.BIOCHEM.67.1.49/CITE/REFWORKS. 9759482

[81] Z. Song , “Roles of the Nucleotide Sugar Transporters (SLC35 Family) in Health and Disease,” Molecular Aspects of Medicine 34, no. 2–3 (2013): 590–600, 10.1016/J.MAM.2012.12.004. 23506892

[82] H. Stöckmann , R. O'Flaherty , B. Adamczyk , R. Saldova , and P. M. Rudd , “Automated, High‐Throughput Serum Glycoprofiling Platform,” Integrative Biology 7, no. 9 (2015): 1026–1032, 10.1039/C5IB00130G. 26189827

[83] S. C. Burleigh , T. van de Laar , C. J. Stroop , et al., “Synergizing Metabolic Flux Analysis and Nucleotide Sugar Metabolism to Understand the Control of Glycosylation of Recombinant Protein in CHO Cells”, BMC Biotechnology 11(1) (2011), 10.1186/1472-6750-11-95. PMC3219575 22008152

