# Supplementary material for: Subcellular Fractionation Enables Assessment of Nucleotide Sugar Donors Inside the Golgi Apparatus as a Prerequisite for Unraveling Culture Impacts on Glycoforms of Antibodies
Source: Biotechnol J. 2025 Mar 24;20(3):e202400678. doi: 10.1002/biot.202400678 (PMC11931351; doi:10.1002/biot.202400678)
Supplement: Supplementary file 1 — Supporting Information [file BIOT-20-e202400678-s001.pdf]

Supplements for: Subcellular fractionation enables Assessment of nucleotide sugar donors inside the Golgi apparatus as a prerequisite for unravelling culture impacts on glycoforms of antibodies

Niklas Regett[1], Marcel Dieterle[1], Fleur Peters1, Max Deuring[1], Kaja Stegmaier[1], Attila Teleki[1], and Ralf Takors[1]

[1] IBVT University of Stuttgart 70569 Stuttgart, Germany, secret@ibvt.uni-stuttgart.de

Keywords: Nucleotide sugar donors, subcellular fractionation, Golgi apparatus, antibody glycosylation, metabolomics

Supplementary Materials

Supplementary Table S 1) Digitonin stock solution for digitonin lysis buffer

Compound	Molarity (mM)	
sodium chloride	115	
sodium bicarbonate	0.02	
digitonin	0.81	
EDTA	2	(protein analysis only)

Supplementary Table S 2) Cell lysis buffer for protein extraction

Compound	Molarity (mM)
sodium chloride	115
sodium bicarbonate	25
Triton X-114	1.97
SDS	0.35
EDTA	2
Natriumazid	0.15

Supplementary Table S 3) Dilution buffer for digitonin lysis buffer

Compound	Molarity (mM)	
Sodium chloride	115	
Sodium bicarbonate	0.02	
EDTA	2	(protein analysis only)

The solutions were adjusted to pH (7.3) and osmolarity (266 mOsmol) to resemble the cultivation medium used according to the manufacturer's recommendations, thereby preventing unnecessary stress on the cells during fractionation.

Supplementary Table S 4) Volumes and compositions of pulsing solutions in NSD-inducing shaking flask pulsings.

		Medium	Fructose	Galactose	Uridine	Manganese (II) chloride dihydrate (Mn)
Pulsing A	Volume [µL]	3698				
	Final conc. [mM]					
Pulsing B	Volume [µL]		3500		128.21	70
	Final conc. [mM]		100		0.5	0.001
Pulsing C	Volume [µL]			3500	128.21	70
	Final conc. [mM]			100	0.5	0.001

Supplementary Table S 5) Literature data for cell and compartment volumes, as well as molar distributions of NSDs between organelles and CP

**Parameter**	**Value**	**Source**
V_{Cell}	1.12×10^{-12} L	[1]
V_{Golgi}	2.50×10^{-14} L (approximately 2.2%)	[1]
$V_{Cytoplasm}^{rel}$	50–60 % (approximately 55%)	[2]
Ratios $[c_{Golgi}]:[c_{Cytoplasm}]$	40:1	
$[c_{Golgi}^{rel}]$	40	[3], [4]
$[c_{Cytoplasm}^{rel}]$	1	

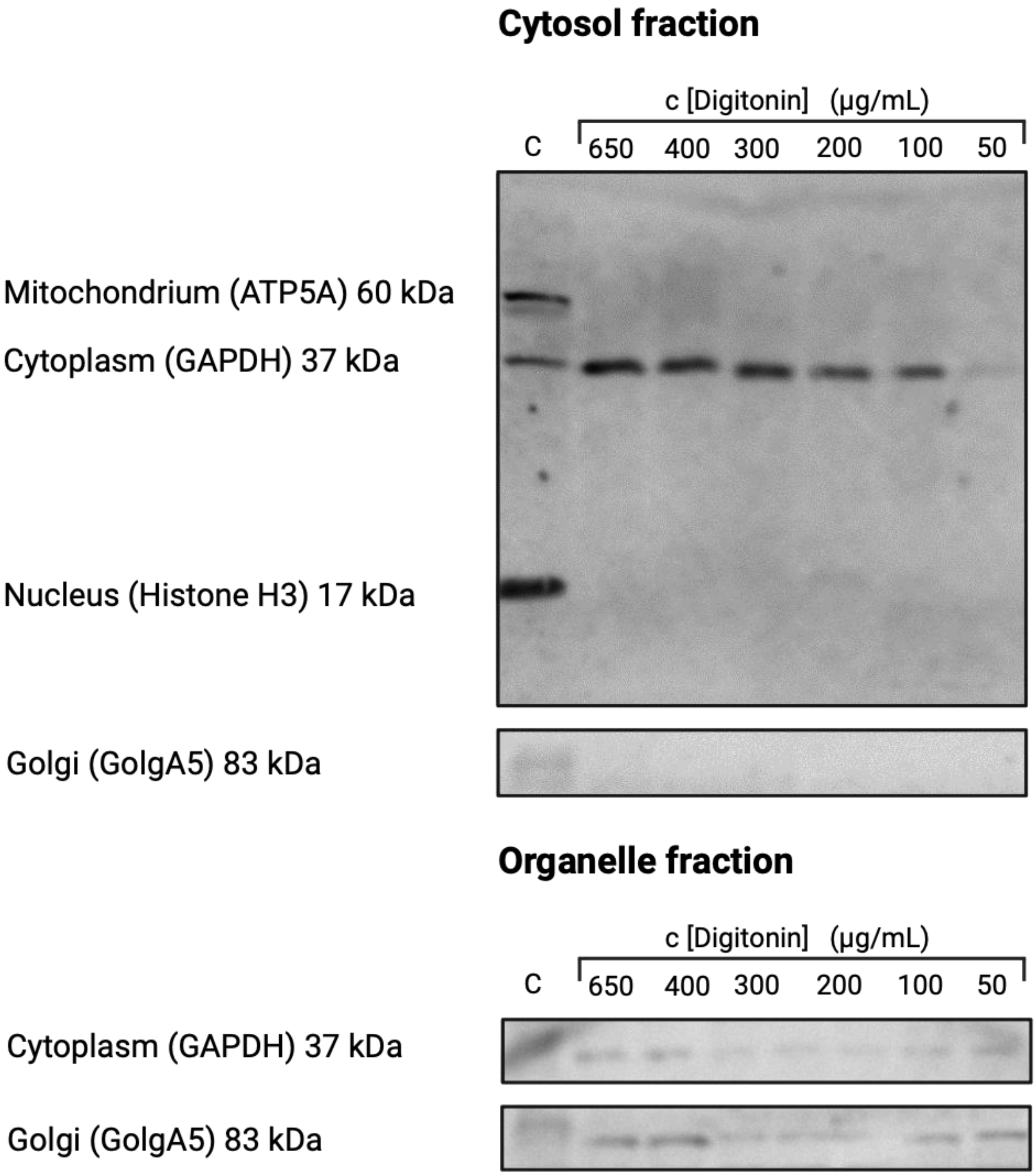

Supplementary Figure S 1) Western blot analysis of protein fractionations. (Top = CP, Bottom= organelle) with marker proteins of different organelles throughout the cell. Created in BioRender. https://BioRender.com/j12b168

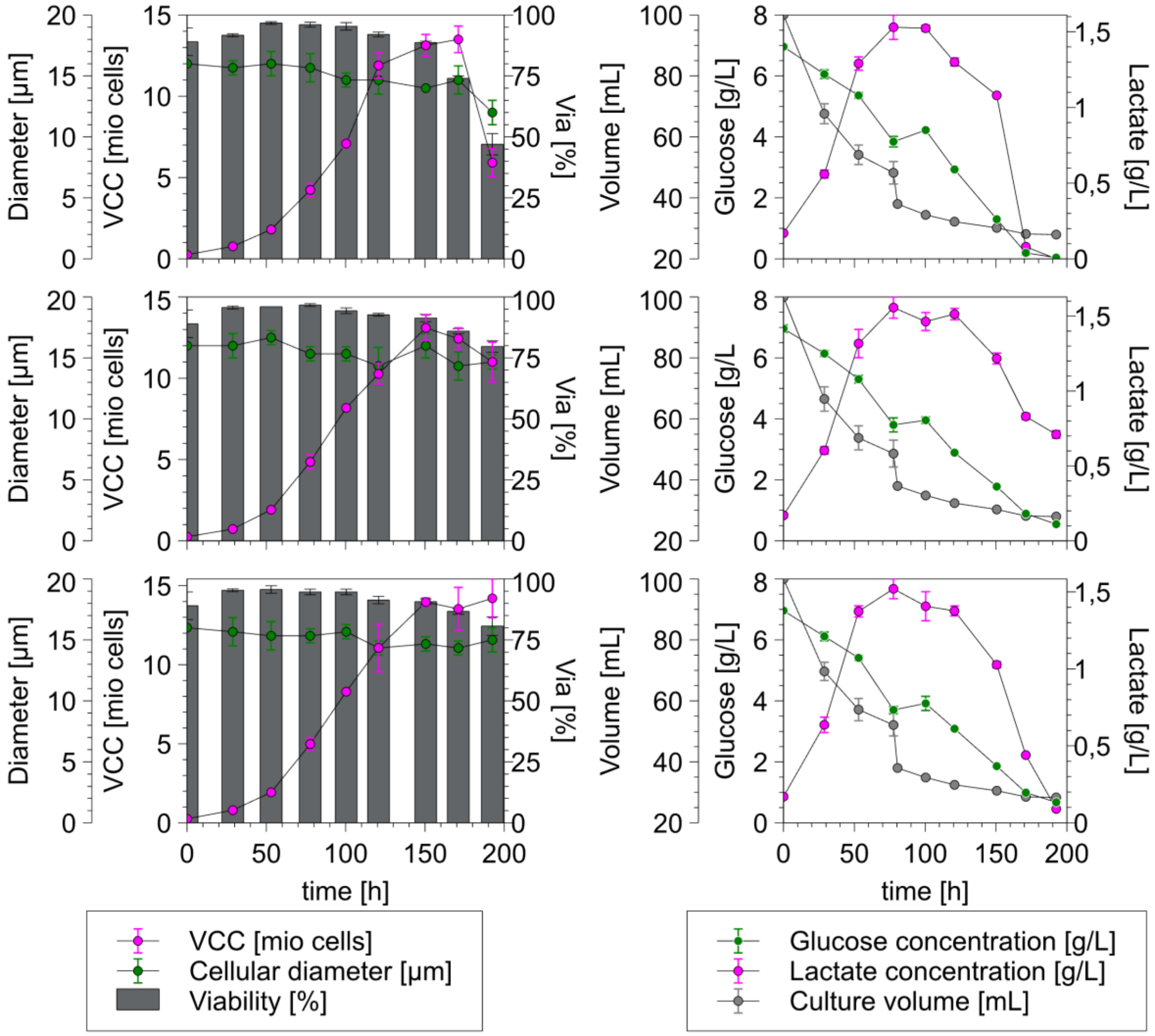

Supplementary Figure S 2) Cultivation parameters of CHO shaking flask cultivations. Parameters under three different nutrient-pulsing regimes. Data show condition (A, Top) with only medium spiked, fructose spiked medium with Mn and uridine (B, middle), and galactose spiked medium with Mn and uridine (C, bottom). Left columns show cell vitality in viable cell count (VCC) [10^6 cells /mL] (pink), viability [%] (grey) and average cell diameter [µm] (green). Right side shows nutrient conditions in substrate concentration (glucose)[g/L] (green), byproduct concentration (lactate) [g/L] (pink) and culture volume [mL] (grey). Error bars show standard deviation for mean values of n=3. In rare cases, sample size had to be reduced.

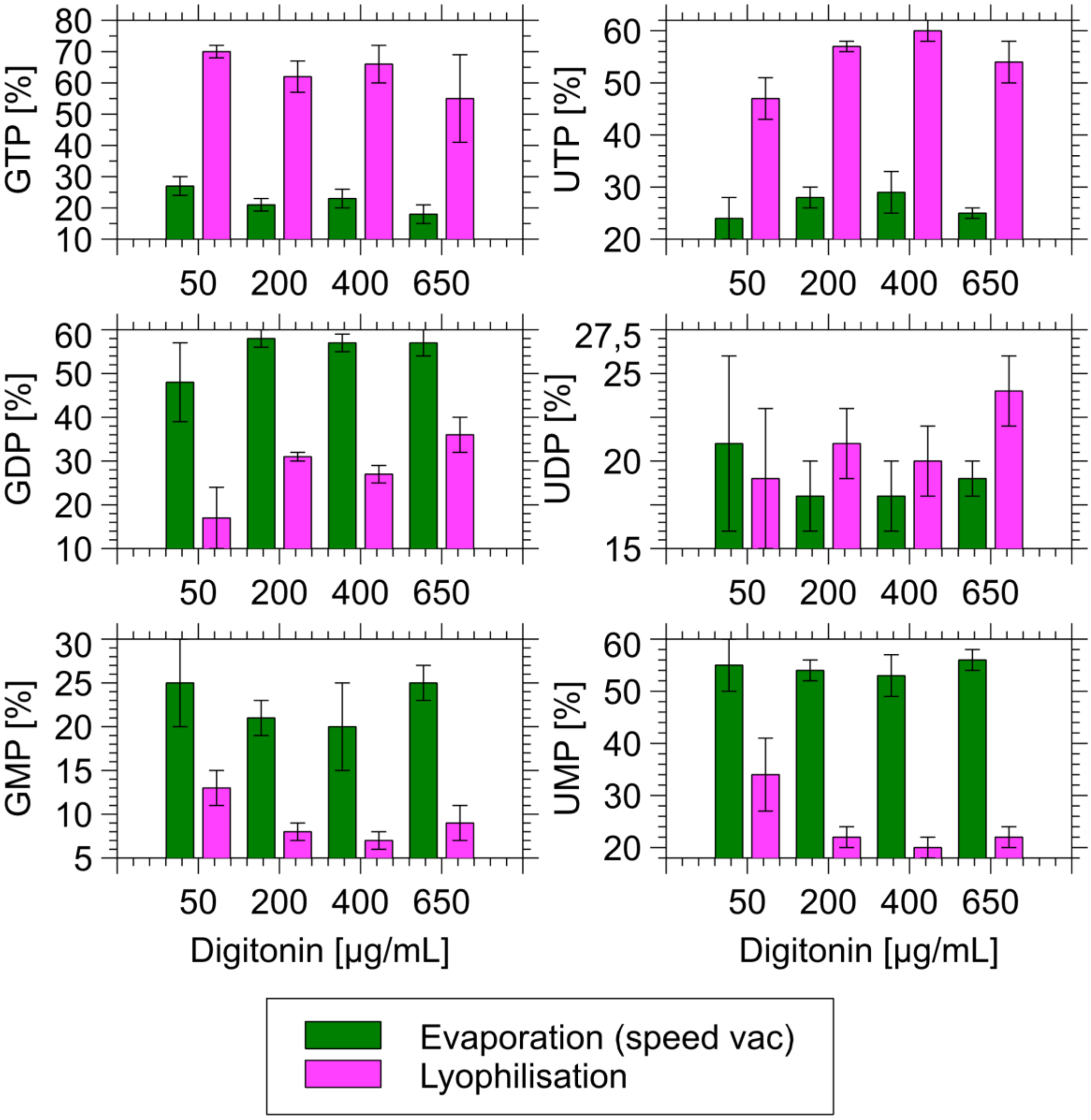

Supplementary Figure S 3) Oligophosphate levels. After concentration of fractions with lyophilization (pink) or evaporation (green). Error bars show standard error for mean values of n=3. In rare cases, sample size had to be reduced to n=2.

Supplementary Table S 6) Mean EC values GEC = guanosine energy charge, UEC = uridine energy charge. Deviations indicate standard deviations of averages of n = 11 with 4 different digitonin concentrations (50 µg/ml, 200 µg/ml, 400 µg/ml and 650 µg/ml) in the fractionation buffer.

	Lyophilization	Evaporation
**GEC**	0.77 ± 0.03	0.5 ± 0.02
**UEC**	0.657 ± 0.06	0.4 ± 0.01

Supplementary Table S 7) Literature values of guanine and uridine energy charges (GEC and UEC) with sources

Energy charge	Literature	Source
GEC	0.82 ± 0.020	[5]
UEC	0.58 ± 0.016	[6]

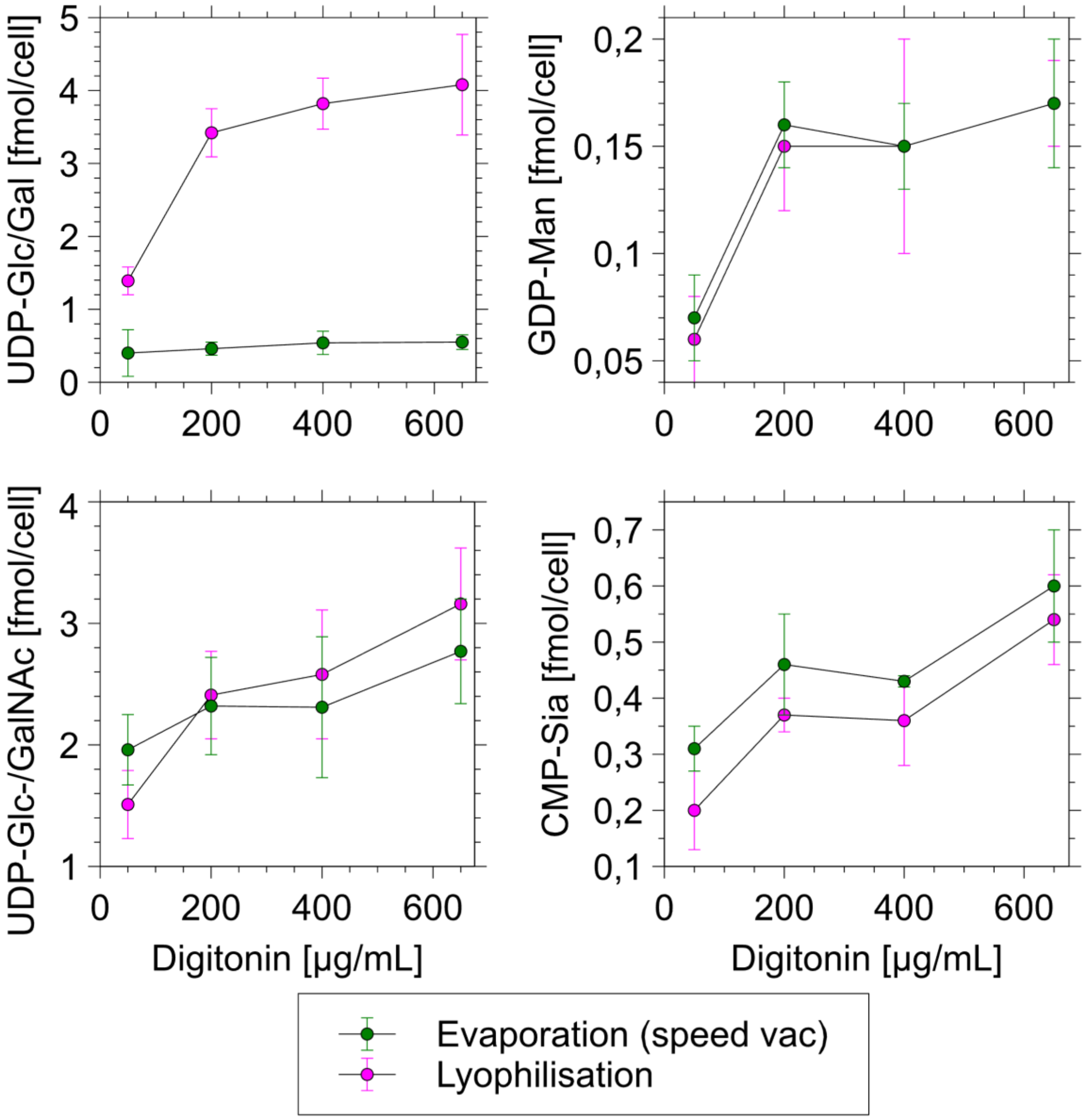

Supplementary Figure S 4) Comparison of NSD conservation in sample concentration. NSD levels after concentration of fractions with lyophilization (pink) or evaporation (green). Error bars show standard error for mean values of n=3. In rare cases, sample size had to be reduced to n=2.

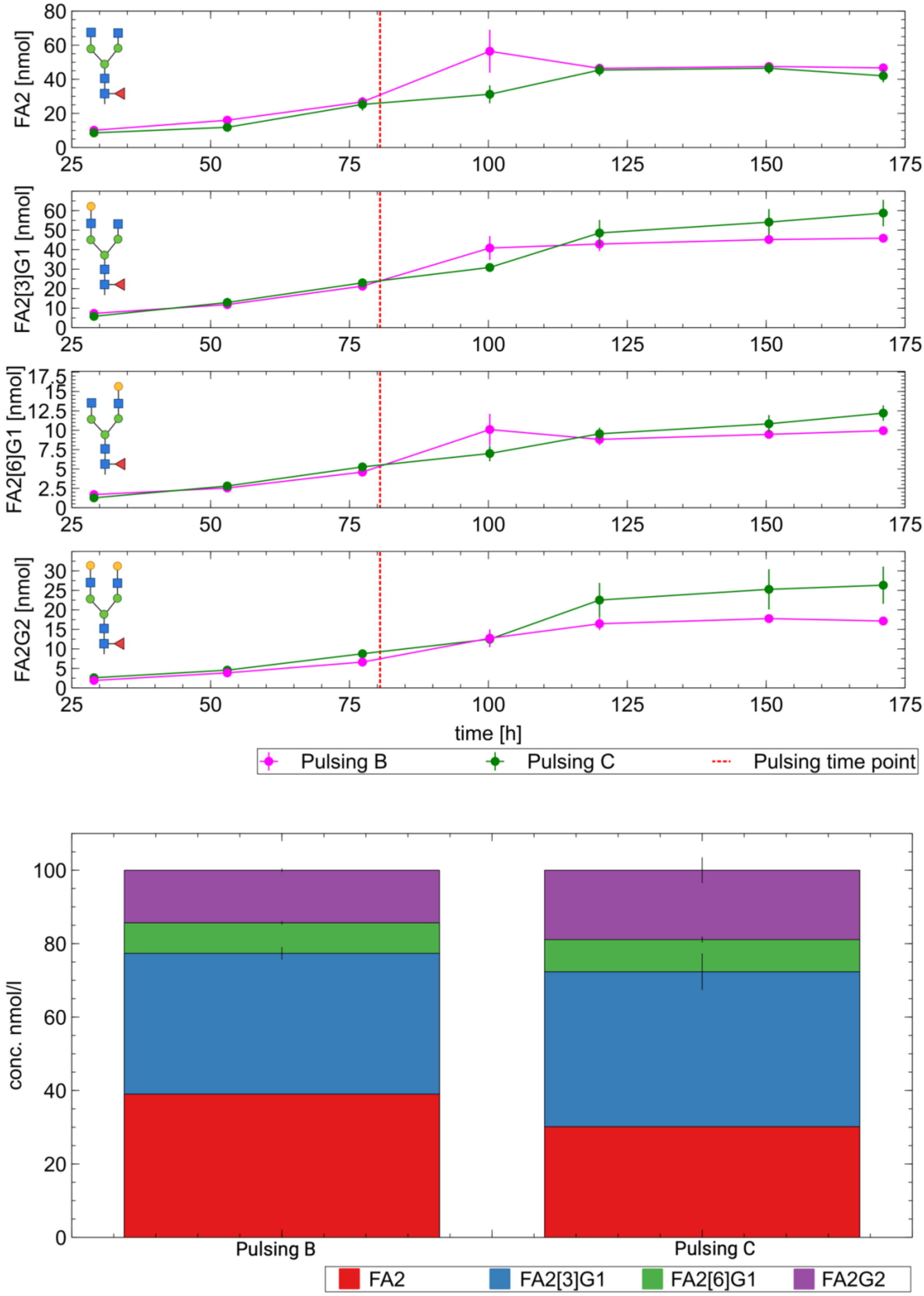

Supplementary Figure S 5) Pulse dependent Glycosylation patterns. Top: Absolute concentration courses of glycans produced under pulsing condition B (fructose, uridine, and Mn, shown with magenta) and C (galactose, uridine, and Mn, shown with green). Bottom: relative glycan distributions expressed on antibodies at end of cultivations under pulsing condition B (left) and C (right). Error bars show standard error for mean values of n=3. In rare cases, sample size had to be reduced. Created in BioRender, https://BioRender.com/b13d335.

[1] P. Kotidis *et al.*, ‘Model-based optimization of antibody galactosylation in CHO cell culture’, *Biotechnol Bioeng*, vol. 116, no. 7, pp. 1612–1626, Jul. 2019, doi: 10.1002/BIT.26960.

[2] B. Alberts, A. Johnson, J. Lewis, M. Raff, K. Roberts, and P. Walter, ‘Molecular biology of the cell, 5th edition by B. Alberts, A. Johnson, J. Lewis, M. Raff, K. Roberts, and P. Walter’, *Biochemistry and Molecular Biology Education*, vol. 36, no. 4, pp. 317–318, Jul. 2008, doi: 10.1002/BMB.20192.

[3] I. Jimenez del Val, J. M. Nagy, and C. Kontoravdi, ‘A dynamic mathematical model for monoclonal antibody N-linked glycosylation and nucleotide sugar donor transport within a maturing Golgi apparatus’, *Biotechnol Prog*, vol. 27, no. 6, pp. 1730–1743, Nov. 2011, doi: 10.1002/BTPR.688.

[4] F. J. Krambeck and M. J. Betenbaugh, ‘A mathematical model of N-linked glycosylation’, *Biotechnol Bioeng*, vol. 92, no. 6, pp. 711–728, Dec. 2005, doi: 10.1002/BIT.20645.

[5] M. Fujita, K. Imadome, T. I.-C. science, and undefined 2017, ‘Metabolic characterization of invaded cells of the pancreatic cancer cell line, PANC-1’, *Wiley Online Library*, vol. 108, no. 5, pp. 961–971, May 2017, doi: 10.1111/cas.13220.

[6] J. S. Hothersall, R. P. Muirhead, C. E. Taylaur, S. Kunjara, and P. Mclean, ‘Changes in Uridine Nucleotides and Uridine Nucleotide Sugars in Diabetic Rat Lens: Implications in Membrane Glycoprotein Formation’, *Biochem Med Metab Biol*, vol. 50, no. 3, pp. 292–300, Dec. 1993, doi: 10.1006/BMMB.1993.1071.
